# Heart failure clinical care analysis uncovers risk reduction opportunities for preserved ejection fraction subtype

**DOI:** 10.1038/s41598-021-97831-1

**Published:** 2021-09-20

**Authors:** Rebecca T. Levinson, Nataraja Sarma Vaitinidin, Eric Farber-Eger, Dan M. Roden, Thomas A. Lasko, Quinn S. Wells, Jonathan D. Mosley

**Affiliations:** 1 Department of Medicine, Vanderbilt University Medical Center, 1285 Medical Research Building IV, Nashville, TN 37232 USA; 2 Internal Medicine II, Heidelberg University Hospital, Heidelberg, Germany; 3 Department of Biomedical Informatics, Vanderbilt University Medical Center, Nashville, TN USA; 4 Department of Pharmacology, Vanderbilt University Medical Center, Nashville, TN USA

**Keywords:** Cardiology, Diseases, Health care, Medical research, Risk factors

## Abstract

Heart failure (HF) has no cure and, for HF with preserved ejection fraction (HFpEF), no life-extending treatments. Defining the clinical epidemiology of HF could facilitate earlier identification of high-risk individuals. We define the clinical epidemiology of HF subtypes (HFpEF and HF with reduced ejection fraction [HFrEF]), identified among 2.7 million individuals receiving routine clinical care. Differences in patterns and rates of accumulation of comorbidities, frequency of hospitalization, use of specialty care, were defined for each HF subtype. Among 28,156 HF cases, 8322 (30%) were HFpEF and 11,677 (42%) were HFrEF. HFpEF was the more prevalent subtype among older women. 177 Phenotypes differentially associated with HFpEF versus HFrEF. HFrEF was more frequently associated with diagnoses related to ischemic cardiac injury while HFpEF was associated more with non-cardiac comorbidities and HF symptoms. These comorbidity patterns were frequently present 3 years prior to a HFpEF diagnosis. HF subtypes demonstrated distinct patterns of clinical co-morbidities and disease progression. For HFpEF, these comorbidities were often non-cardiac and manifested prior to the onset of a HF diagnosis. Recognizing these comorbidity patterns, along the care continuum, may present a window of opportunity to identify individuals at risk for developing incident HFpEF.

## Introduction

Heart failure (HF), a clinical syndrome driven by the heart’s inability to meet the oxygen needs of the body, is a significant cause of annually increasing morbidity and mortality. HF accounted for 1 in 8 deaths in the U.S. in 2017^1^. In 2018, there were about 6.2 million adults with heart failure in the United States, and 379,800 death certificates listed heart failure (13.4%)^2^. Heart failure is broadly subtyped by left ventricular ejection fraction (LVEF) into HF with reduced ejection fraction (HFrEF, LVEF < 40) and HF with preserved ejection fraction (HFpEF, LVEF ≥ 50)^3^. Currently, HFpEF patients account for about 50% of all heart failure patients^3,4^. There is no cure for HF and, for HFpEF, there are currently no life-extending treatments^3–7^. Hence, prevention is paramount for reducing the burden of HF, especially for HFpEF, which is becoming the more prevalent subtype^3–7^.

Identifying high risk individuals is essential for effective prevention, and this requires a clear understanding of the evolving epidemiology of HF^8–11^. In particular, a clearer understanding of the spectrum and temporality of the clinical manifestations of early HF could better enable primary care providers to identify high risk patients with sufficient lead time to enable early intervention to delay or prevent the onset of HF and sequelae^12,13^. Defining the relevant clinical epidemiology requires large, contemporary clinical cohorts, such as those who seek care at large medical centers^14–20^.

We leveraged electronic health records (EHR) from ~ 2.7 million individuals seeking routine clinical care at Vanderbilt University Medical Center. We identified a large collection of HF cases and characterized the comorbidity profiles of HF subtypes prior to and at the time of their clinical diagnosis. We asked if there were distinct longitudinal patterns of comorbidity accumulation between the HF subtypes, as patients moved through the healthcare system, and if this could have implications for HF management.

## Methods

All individuals were derived from Vanderbilt University Medical Center’s Synthetic Derivative database, a research tool for conducting epidemiological studies using de-identified EHR data, especially suited to work with tools of machine learning and big data^21–24^. This resource comprises inpatient and outpatient clinical data from multiple sources including diagnostic and procedure codes (ICD-9 [International Classification of Disease, Ninth revision] and CPT [Current Procedural Terminology]), demographics, text from clinical notes, laboratory values, procedural reports (e.g., echocardiograms), and medications extracted individual clinical records^23–25^. The study was reviewed and approved by Vanderbilt’s Institutional Review Board and was determined to be non-human subjects research. These data contain no HIPAA or other personal identifiers.

### Clinical phenotypes

Medication data were extracted using the validated MEDEX tool^26^. Keyword features were extracted from source documents (problem lists and clinical documents) and occurrences were excluded if a negation term (such as “not” or “ruled out”) was within 100 characters of the keyword (Supplementary Tables 1 and 2)^27,28^. For each individual, sex, race (white, black or other), blood pressure, heart rate, body mass index (BMI), BNP values, and clinician visit dates and types (i.e., inpatient/outpatient, clinic type) were extracted from structured tables in the SD. Diagnoses of myocardial infarction (MI), coronary artery disease (CAD), hypertension, dyslipidemia, atrial fibrillation (AF), type 2 diabetes (T2D), and chronic kidney disease (CKD) were based on previously validated EHR algorithms (Supplementary Table 3)^29^.

### Echocardiographic measures of cardiac structure and function

Measures, extracted from transthoracic echocardiography (TTE) reports, using previously described approaches^29,30^, included left ventricular wall thickness, left ventricular ejection fraction (LVEF), diastolic function, and cardiac chamber dimensions. For each HF case with one or more LVEF measurements, the following HF subtypes were defined:

1. HFpEF: All LVEF measurements ≥ 50%.
2. HFmEF (Heart Failure with Mid-Range Ejection Fraction): Any LVEF < 50% and no LVEF < 40%
3. HFrEF: Any LVEF ≤ 40%.

### PheWAS phenotypes

Phenome-wide association study (PheWAS) codes are validated groupings of related ICD-9 billing codes that capture the extended range of clinical diagnoses within an EHR data set^31,32^. A complete list of PheWAS codes and their mappings to ICD-9 codes can be found at https://www.vumc.org/cpm/cpm-blog/phewas-phenome-wide-association-studies. For a given PheWAS code, cases are individuals with 1 or more codes and controls are individuals with no related codes. After excluding phenotypes affecting a single sex or with ≤ 100 cases, 1224 clinical phenotypes were included in analyses.

### Universal definition of heart failure

We examined how many of the cases used in the Validation set met the Bozkurt et al.^20^ universal heart failure algorithm criteria based on the features available to the machine learning algorithm. In line with the guidelines, we tested for the presence of any of the following symptoms: pulmonary edema, pleural effusion, orthopnea, paroxysmal nocturnal dyspnea, nocturnal cough, lower extremity edema, dyspnea on exertion, jugular venous distension/elevated jugular venous pressure/elevated JVP, cardiomegaly, third heart sound, hepatomegaly, rales, ejection fraction < 50%, and an outpatient NT-Pro-BNP, pg/ml ≥ 125. The proportion of individuals who met all of these criteria was determined.

### Analysis

The random forest machine learning classifier, implemented in *ScikitLearn* (v0.18.1)^33^, was used to identify heart failure cases^34^. The approach and implementation are detailed in the Supplementary Methods. In brief, the machine learning model was developed on a training set (n = 1091) and validated on a testing set (n = 468). Based on the final set of features selected, an independent testing set demonstrated that the model had a positive predictive value (PPV) of 0.92 and a case sensitivity and specificity of 0.67 and 0.99, respectively, as compared to manual clinical record review by a cardiologist (Supplementary Tables 4 and 5). This validated model was then deployed in the EHR population to identify heart failure cases. For cases with TTE measurements, heart failure subtypes were assigned based on LVEF (see above). While a manual record review by a cardiologist confirmed the heart failure diagnosis among all cases used in the Validation set, only 34.8% met the Universal Definition of HF (described above) based on features employed by the random forest classifier.

Descriptive statistics were generated for all HF cases as well as for each subtype (HFpEF, HFrEF, HFmEF) separately. Between group differences were assessed using the Mann–Whitney U test or Chi-square test for continuous and dichotomous variables, respectively.

We also conducted stratified analyses comparing HF cases identified before and after 2005, the approximate midpoint of the time range for which EHR data were available, to characterize secular trends in HF epidemiology. Subjects were classified by LVEF in 10-year age ranges, centered around decades for which LVEF data were present, and stratified by sex and date of HF diagnosis.

PheWAS analyses were used to comprehensively scan the medical phenome to identify clinical diagnoses differentially associated with HFrEF versus HFpEF, since these subtypes had the largest numbers of individuals. Because PheWAS phenotypes are highly correlated, we conducted a 2-step analysis to identify phenotypes independently associated with the HF subtypes. First, multivariable logistic regression analysis adjusting for age at HF diagnosis, sex, and self-reported race was used to test the association between each PheWAS code and the HF subtype (odds-ratios are the association with HFpEF, as compared to HFrEF). Phenotypes with a Benjamini–Hochberg (B–H) false discovery rate (FDR)^35^ q-value < 0.1 were then jointly analyzed using a multivariable logistic model in conjunction with a stepwise selection feature (using Proc Logistic in SAS) that retained all phenotypes with an independent association p < 0.05. PheWAS analyses were stratified by 10-year age ranges.

The ML algorithm also assigned a diagnoses date for each case. To better define the clinical events leading up to a HF diagnosis, we extracted ICD-9 based diagnoses during each of the three years prior to diagnosis of HF and mapped them to corresponding PheWAS phenotypes. Because ascertainment of diagnoses during this time may be incomplete in individuals referred for specialty care, analyses were limited to those individuals receiving medical care at VUMC prior to diagnosis of HF, defined as two or more primary care or cardiology outpatient visits over the three-year period. Descriptive statistics were generated for PheWAS diagnoses and medical encounters prior to HF diagnosis for both HFpEF and HFrEF subjects and were also stratified by 10-year age groups.

## Results

**Figure 1:**
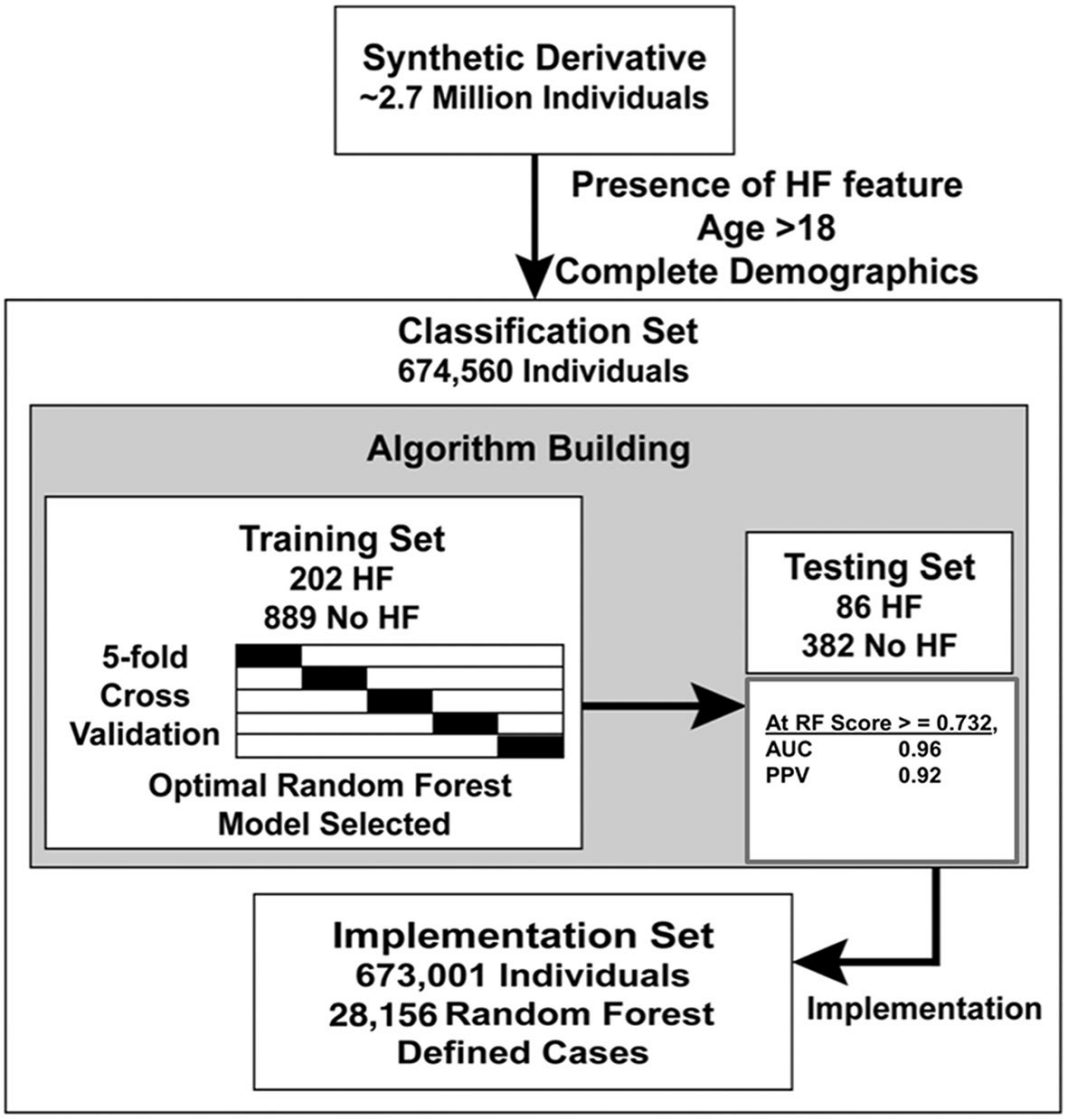
Overview of case identification and algorithm development. Individual level data were derived from an EHR comprising 2.7 million records. The machine learning algorithm was trained and tested on manually adjudicated sets of HF cases and non-cases. The final algorithm was deployed in an Implementation set to identify HF cases across the EHR. Performance measures for the HF classifier is shown for the Testing Set.

The ML classifier identified 28,156 HF cases (Fig. 1). Of these, 8322 (30%) were classified as HFpEF, 11,677 (42%) as HFrEF, 1958 (7%) HFmEF, and 21% had no LVEF data. HFrEF cases were more likely to be male (66 vs 42%) and have coronary heart disease (78 vs. 63%), as compared to HFpEF cases, (Table 1). Additional results on the ML model are presented in the Supplementary Methods. A significant portion of the comorbidities presented in Table 1 include diagnoses associated after a HF diagnosis, the fractions before and after HF diagnosis are presented in Supplementary Table 6. Many of the comorbidities appeared after a heart failure diagnosis. Conversely, subjects with HFpEF, were older (67 vs 62 years), had higher BMI’s (30.7 [interquartile range, 25.9, 36.9] vs 28.2 [24.6, 33.0] kg/m^2^), and had a higher prevalence of hypertension (91% vs 87%) (p < 1 × 10^–15^ for all). Subjects with HFmEF variably differed with respect to HFpEF and HFrEF for these features.

**Table 1:** Characteristics of all HF cases and subtypes.

Characteristic*	All HF cases	HFpEF	HFmEF	HFrEF
	(n = 28,156)	(n = 8322)	(n = 1958)	(n = 11,677)
Male sex [n(%)]	15,760 (56.0)	3488 (41.9)	1102 (56.3)	7706 (66.0)
**Race/ethnicity [n(%)]**				
Black	4239 (15.1)	1314 (15.8)	267 (13.6)	1941 (16.6)
Other	1816 (11.5)	394 (4.7)	81 (7.4)	663 (5.7)
White	22,101 (78.5)	6614 (79.5)	1610 (82.2)	9073 (77.7)
Age last ICD9 code (years)	69.1 [59.0, 78.5]	71.1 [60.5, 80.5]	70.2 [59.7, 79.5]	66.6 [56.6, 76.0]
Age of HF assignment	65.0 [54.6, 74.6]	66.7 [56.0, 76.3]	65.3 [54.7, 75.4]	61.9 [51.8, 71.3]
Length of record (years)	5.5 [1.5, 10.8]	6.8 [2.5, 12.4]	7.0 [2.8, 12.5]	5.9 [2.0, 11.2]
BP systolic	121.0 [111.0, 132.0]	125.0 [116.0, 135.1]	122.0 [114.0, 132.6]	117.0 [107.5, 127.0]
BP diastolic	66.0 [60.0, 72.0]	65.8 [60.0, 72.0]	66.0 [60.0, 72.0]	66.0 [60.0, 72.0]
Heart rate	77.0 [70.0, 85.0]	76.0 [69.0, 85.0]	75.0 [68.4, 84.0]	77.8 [70.0, 86.0]
BMI kg/m^2^	29.3 [25.0, 35.0]	30.7 [25.9, 36.9]	29.1 [24.8, 34.3]	28.2 [24.6, 33.0]
Myocardial infarction [n(%)]	6633 (23.6)	1519 (18.3)	563 (28.8)	3832 (32.8)
Coronary artery disease [n(%)]	19,104 (67.9)	5206 (62.6)	1431 (73.1)	8958 (76.7)
Hypertension [n(%)]	24,563 (87.2)	7600 (91.3)	1792 (91.5)	10,202 (87.4)
Dyslipidemia [n(%)]	22,906 (81.4)	6754 (81.2)	1696 (86.6)	10,064 (86.2)
Type 2 diabetes [n(%)]	6163 (21.9)	1951 (23.4)	500 (25.5)	2653 (22.7)
Atrial fibrillation [n(%)]	12,892 (45.8)	3978 (47.8)	1002 (51.2)	5704 (48.8)
Chronic kidney disease [n(%)]	9121 (32.4)	2945 (35.4)	673 (34.4)	4291 (36.7)
Echocardiography present [n(%)]	21,957 (78.0)	8322 (100.0)	1958 (100.0)	11,677 (100.0)
Lowest EF value	40.0 [20.0, 55.0]	55.0 [55.0, 55.0]	45.0 [42.5, 45.0]	22.0 [15.0, 30.0]
BNP measure present [n(%)]	16,249 (57.7)	5352 (64.3)	1284 (65.6)	7677 (65.7)
BNP lifetime (pg/ml)	366.5 [146.0, 869.0]	275.3 [110.0, 619.5]	320.0 [128.0, 721.0]	483.0 [199.0, 1142.0]

*Median and inter-quartile range (IQR) or N (%) as appropriate. Comorbidities may have been diagnosed after the HF diagnosis.

**Figure 2:**
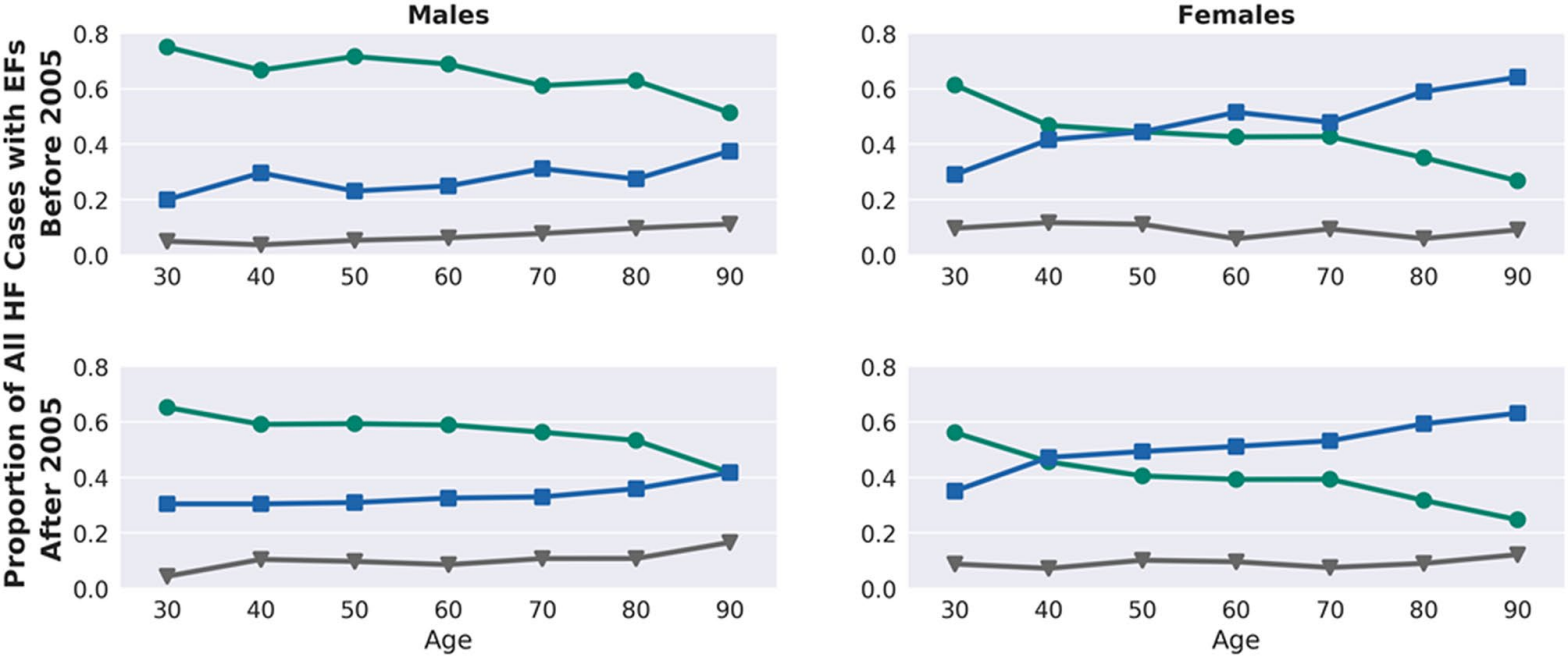
Relative proportions of HF subtypes by age, sex and time period. The proportions of HF subtypes amongst all HF subjects with available EF measurements by age decade. Individuals are stratified by sex and year of HF diagnosis (before or after 2005). The green line indicates HFrEF, the blue line HFpEF, and the grey line HFmEF. Individuals with longer medical records may contribute to multiple age bins.

HF cases entered the medical system between 1989 and 2017, with the majority entering after the 2005 (the middle timepoint) [N = 19,475 (69%)]. The relative proportions of HF subtypes, by age and sex, were similar before and after 2005 (Fig. 2). Among both sexes, the proportion of individuals with HFrEF decreased with age. Among men, HFrEF was the most common subtype, though the relative proportion of HFrEF decreased in more recent time periods and with increasing age. Among women, HFpEF was the more prevalent subtype after age 30–40 and this proportion increased with age. Compared to HF cases diagnosed before 2005, subjects entering the health system after 2005 were older (66.0 vs 62.5 years), more likely to be diagnosed with hypertension (89% vs 83%), diabetes (24% vs 18%), CKD (35% vs 28%), and AF (47% vs 43%) (p < 1 × 10^–15^ for all) (Supplementary Table 6A). The trend in increasing age and diagnoses of hypertension, T2D, dyslipidemia, and CKD was seen across all subclasses. The prevalence of CAD remained similar in all HF subclasses, and median BMI was above 28 and 30 for HFrEF and HFpEF, respectively. Among HFrEF subjects, those entering the medical system after 2005, were more likely to have a history of myocardial infarction (MI) (34% vs 30%; p = 1.96 × 10^–8^) and a slightly higher LVEF nadir (25% vs 20%; p = 1 × 10^–15^).

**Figure 3:**
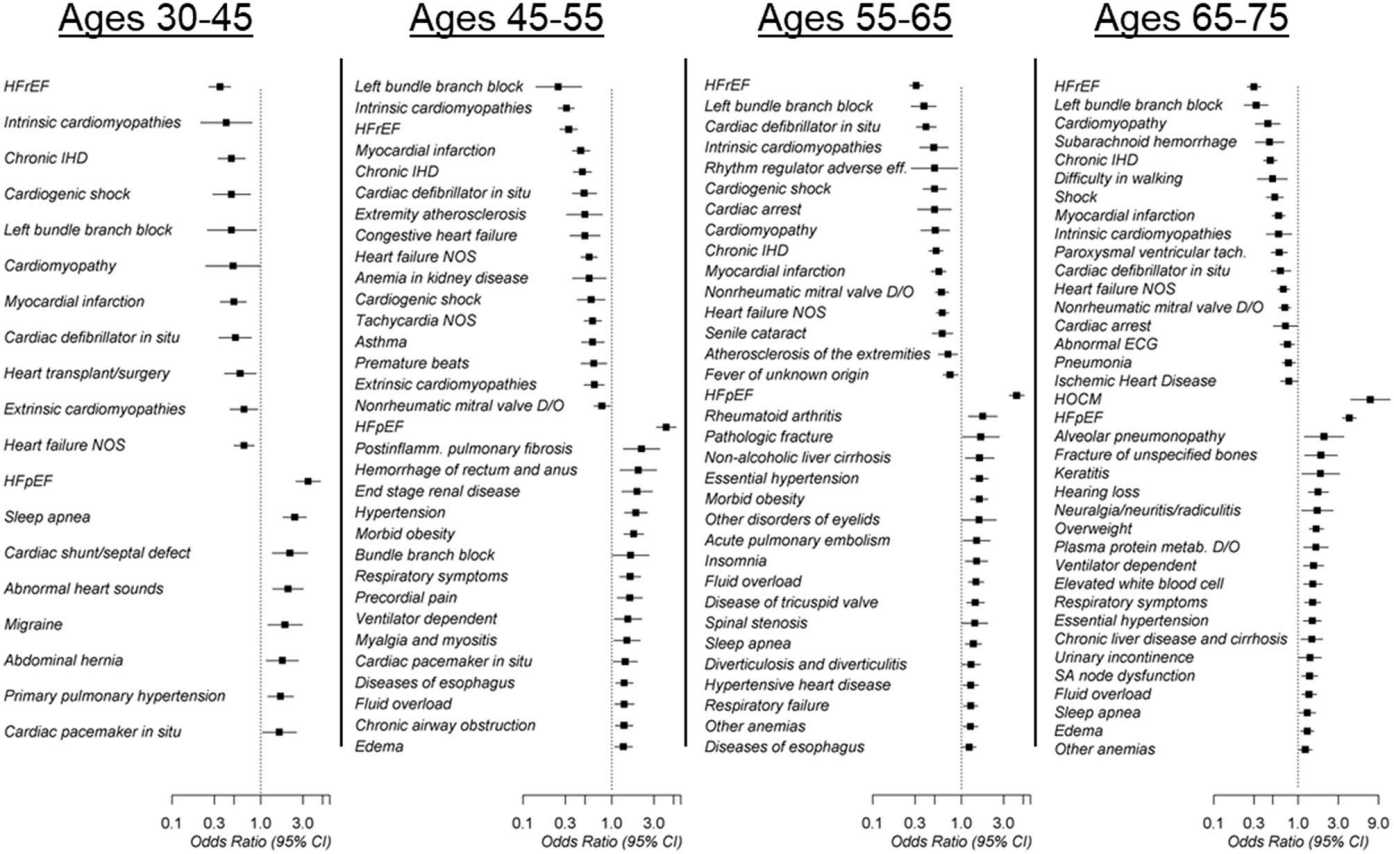
Summary of clinical phenotypes associated with HF subtypes, by age group. A forward selection logistic regression model adjusting for age, sex and race was used to identify clinical phenotypes independently associated with HFpEF, versus HFrEF. An odds-ratio (OR) < 1 indicates that the phenotype is more prevalent among individuals with HFrEF and an OR > 1 indicates the phenotypes more prevalent with HFpEF.

Phenome-wide comparisons identified clinical diagnoses differentially associated with HFpEF versus HFrEF cases across multiple age ranges. Among all age strata, there was a directionally consistent association between an ICD-9 based diagnosis of a heart failure subtype (HFpEF or HFrEF) and the assigned ML subtype (Fig. 3). For instance, individuals age 30–45 with HFpEF were less likely to carry a clinical diagnosis of HFrEF (odds-ratio [OR] = 0.35, 95% confidence interval [0.27–0.47], p = 2.9 × 10^–14^) more likely to have diagnosis of HFpEF (OR = 3.52 [2.54–4.88], p = 3.4 × 10^–14^) (Supplementary Table 7).

Across all ages, most diagnoses prevalent among HFrEF cases were related to the circulatory system (Supplementary Fig. 1). HFrEF was consistently associated with ischemic heart disease (IHD) and MI across age groups and was associated with valve disease and sequelae of IHD in older age groups, including ventricular arrhythmias and ECG abnormalities such as left bundle branch block (Fig. 3 and Supplementary Fig. 2).

In contrast, diseases associated with HFpEF cases were more heterogeneous and reflected a higher burden of comorbidities across the clinical disease spectrum. Among these were diagnoses related to symptomatic HF, such as respiratory failure, volume overload and edema, as well as obesity and related complications. For example, HFpEF was associated with sleep apnea at all ages, a diagnosis of obesity after age 45, and non-alcoholic liver disease after age 55 (OR = 1.59 [1.08–2.32], p = 1.8 × 10^–2^). It was also associated with a diagnosis of hypertrophic obstructive cardiomyopathy among individuals ages 65–75 (OR = 6.37 [3.81–10.7], p = 1.9 × 10^–12^).

In the youngest age group, the patterns of comorbidities were suggestive of HF etiologies secondary to congenital heart disease, extra-cardiac etiologies (e.g., pulmonary hypertension), as well as metabolic risk factors (Fig. 3, Supplemental Fig. 3).

**Figure 4:**
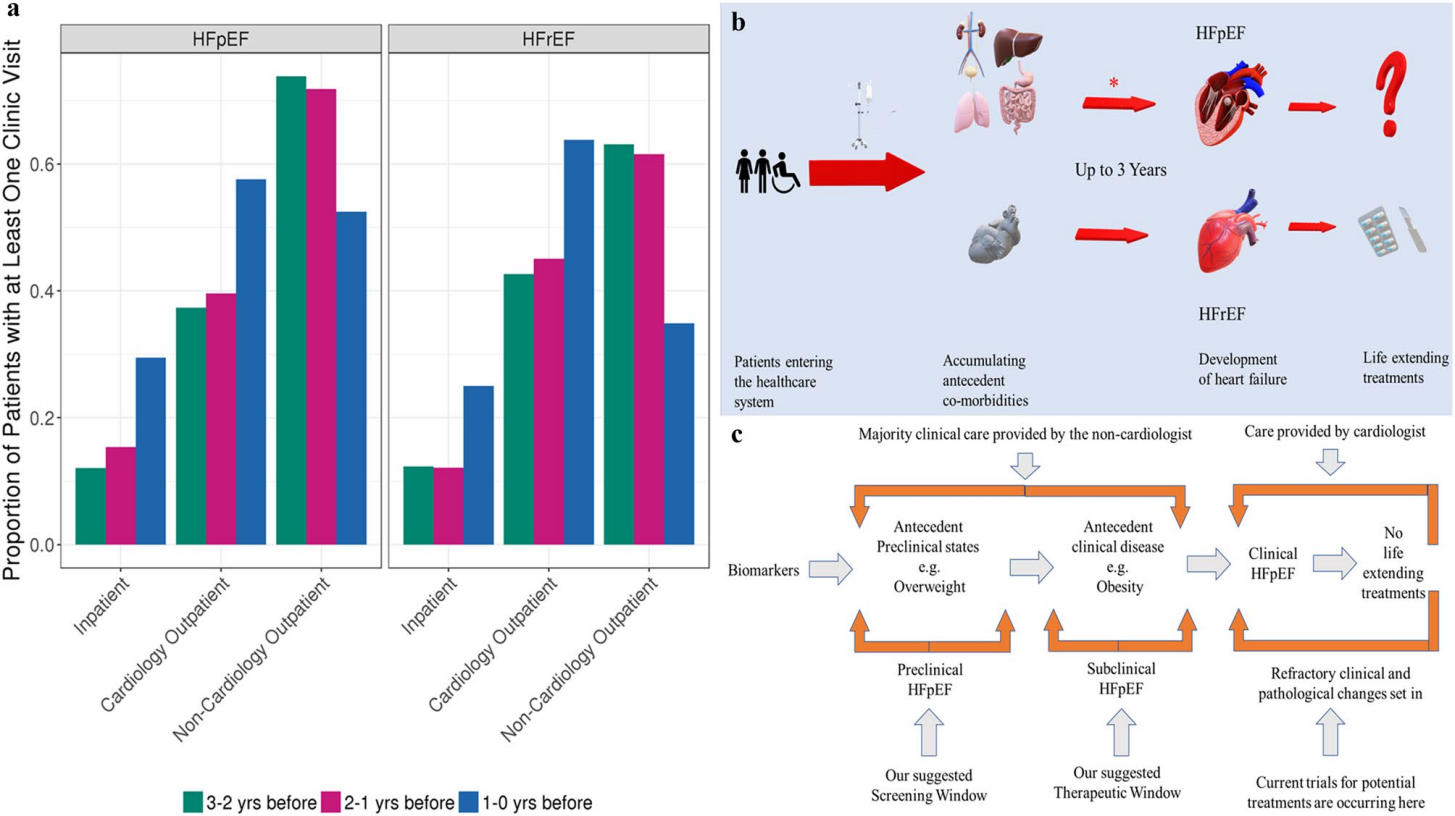
(**a**) Proportion of HFpEF and HFrEF patients who had inpatient, outpatient cardiology, or outpatient non-cardiology encounters in the three years prior to HF. The proportion of HFpEF and HFrEF cases who meet medical home criteria (3 visits in 5 years) and have clinical encounters with in different clinical settings. (**b**) Intersection of the care continuum and the development of heart failure. Individuals accumulate comorbidities, for up to 3 years, as they progress through the hospital system receiving care, before a diagnosis of HF. This phenome-wide study demonstrates that non-cardiac antecedent comorbidities preferentially associate with the development of heart failure with preserved ejection fraction (HFpEF), as compared to heart failure with reduced EF (HFrEF). (**c**) The layered continuum of HFpEF. HFpEF is a complex**,** layered continuum of varied etiologies that, over-time, converge at the physiological endpoint of a “stiff heart”, as the most apparent clinical feature. The antecedent period may represent the preclinical and subclinical forms of HFpEF, where potential screening and therapeutic opportunities might exist.

Hospitalizations and cardiovascular evaluations became increasingly common in the years immediately prior to HF diagnosis (Fig. 4). In the year immediately preceding a HF diagnosis, approximately one quarter of both HFpEF and HFrEF subjects were hospitalized and over half were evaluated in outpatient cardiology clinics. During the three years prior to diagnosis subjects were increasingly diagnosed with cardiovascular comorbidities including IHD, hypertension, dyslipidemia, diabetes, and coronary atherosclerosis as well as symptoms such as shortness of breath, edema, and chest pain (Supplementary Fig. 3).

Overall patterns were similar between HFpEF and HFrEF, though IHD was more common in HFrEF and hypertension more common in HFpEF. HFpEF subjects also accumulated comorbid diagnoses for a slightly longer duration before a HF diagnosis, as compared to subjects with HFrEF (~ 2 years vs ~ 1 year). We examined the timing of the first mention of diuretic use, as a marker of early symptomatic heart failure, in the 3 years prior to a HF diagnosis. For HFpEF and HFrEF, respectively, 98% and 99% of loop diuretics were first mentioned only within the 6 months prior to a diagnosis (Supplementary Fig. 7, Supplementary Table 8).

## Discussion

This paper defined the comorbidity profiles and patterns of healthcare utilization for a large collection of HF cases receiving routine healthcare. There were clear differences in the profiles for the HF subtypes; overall, HFpEF-associated comorbidities were less likely to be cardiac diseases, as compared to HFrEF comorbidities (summarized in Fig. 4). Many of these comorbidities were present for up to 3 years before a diagnosis of HF was made. For HFpEF, these comorbidities were often presentations of symptomatic HF as well as complications often attributable to obesity. Recognition of these comorbidity patterns may present a window of opportunity to identify individuals at risk for developing incident HFpEF prior to fulminant disease.

Our rationale for pursuing these studies was motivated by the need to provide a fuller understanding of mechanisms contributing to HF morbidity, especially HFpEF, for which there are no proven life-extending therapies. The 2020 National Heart, Lung, and Blood Institute Working Group Summary on HFpEF research priorities has proposed potential molecular mechanisms of cardiac re-modelling in HFpEF attributable to comorbidities^6^. In particular, non-cardiac mechanisms underlying HF development need more research, and successful implementation of therapies targeting these mechanisms requires delineation of the presence and timing of these comorbidities in the context of the natural history of the disease^4^.

The timing and nature of the comorbidities associated with HFpEF was indicative of a frequently insidious disease course characterized by an accumulation of diagnoses related to heart failure symptomology and volume overload such as edema, respiratory symptoms and “fluid overload”. There were also diagnoses indicative of organ damage due to poorly controlled hypertension, such as hypertensive heart disease. Other associations were indicative of advance disease, such as anemia^36^, and chronic conditions that are downstream sequelae of chronic heart disease such as hearing loss^37^. The comorbid association patterns among 30–45-year-olds also highlight mechanisms particular to the development of HF symptoms in this age group that are not classically associated with the heart failure syndrome. These include congenital heart defects and primary pulmonary hypertension, though the latter is often misclassified in EHR data sets and typically represents secondary pulmonary hypertension. Thus, this age group is likely enriched in HF subtypes not representative of the more common forms of HF seen in older adults.

HFpEF was also associated with a collection of diagnoses related to chronic obesity including morbid obesity, sleep apnea and non-alcoholic liver cirrhosis, highlighting the significant contribution of obesity to adverse LV remodeling, including diastolic dysfunction^38^. There was a temporality to these associations. For instance, sleep apnea was more common at a younger age, suggesting that this may be an important early clinical biomarker of risk for future HFpEF risk. In contrast, older ages were associated with the sequelae of end-organ failure, including non-alcoholic liver disease and HFpEF. It has been demonstrated that obesity is associated with an increased natriuretic peptide receptor type C/type A ratio. It has been postulated that this altered ratio may cause breakdown and deficiency of natriuretic peptide, leading to impaired cardiac function and possible progression to HFpEF^4,6^.

In contrast to HFpEF, HFrEF was associated with cardiovascular conditions that adversely impacted left ventricular systolic function. These associations included intrinsic (e.g., idiopathic) cardiomyopathies among individuals < 55 years old, structural heart disease (e.g., valve disease) at older ages, and ischemic heart disease across all age ranges. HFmEF patients in our study, comprising 10% of the HF cases, were a hybrid of HFpEF (female, HTN, DM2), and HFrEF (CAD burden) features, in agreement with prior observations^39^.

Our overall findings are corroborated by a recent study that examined the burden of 15 comorbidities among participants in the Atherosclerosis Risk In Communities (ARIC) study diagnosed with acute, decompensated heart failure^40^. In that study, HFpEF had a higher average burden of comorbidities, as compared to HFrEF. Importantly, a higher comorbidity burden was associated with higher mortality rates. Our study extends that by demonstrating that the higher comorbidity burden extends to a far broader spectrum of comorbidities. We also add to their findings by demonstrating that these comorbidities are antecedent in nature, are along the care continuum, and may carry a differential risk for developing incident HFpEF.

The majority of encounters occurring prior to a HF diagnosis were with non-cardiologist providers, especially for patients who went on to develop HFpEF. Approximately 50% of HFpEF patients received loop diuretics before their HF diagnosis, and most (98%) received them in the 6 months prior to HF diagnosis. Sensitizing primary providers that a loop diuretic requirement may suggest the onset of symptomatic heart failure could advance the lead time for a HF diagnosis and potentially enhance outcomes.

A large portion of research related to HFpEF has focused on adverse cardiac remodeling including diastolic dysfunction, cardiac hypertrophy and myocardial fibrosis^4^. Less research focuses on the multi-organ system processes that may play a role in the development of HFpEF. Our findings support the notion that HFpEF is a layered continuum characterized by premorbid states, such as obesity, that ultimately can lead to HFpEF (Fig. 4). This period of premorbid conditions may represent preclinical HFpEF, a timepoint where biomarkers like natriuretic peptide may be informative for early risk stratification. Most therapeutic trials recruit participants with fulminant HFpEF, a timepoint where refractory pathological changes have likely set in^3–7^. Better characterization of the premorbid state may identify timepoints where therapies may demonstrate greater benefit.

There are several novel aspects of the current study. The combination of a large data set, a broad representation of cardiac and non-cardiac phenotypes and large numbers of individuals across the full adult age spectrum provided an opportunity to identify important drivers of HF across the life course. In the younger age group, ischemic heart disease was a less common HF comorbidity, as compared to older ages. Cardiometabolic risk factors were common among this age group, and structural heart disease related to congenital heart defects were also more prevalent. Across all ages, HFpEF is a condition associated with a broad range of comorbidities, many of which are related to obesity. Prior to a HF diagnosis, most clinical encounters were with non-cardiologists for both HF subtypes, especially HFpEF. Importantly, the methods used here are easily portable to other EHR environments, and implementation of this approach across a broad range of EHR data sets will enable efficient, contemporaneous characterization of the evolving epidemiology of HF and will highlight important differences across diverse populations which will directed enhance treatment and prevention.

There are limitations to this study. The VUMC EHR is an observational, single site data set, which can be associated with differentially missing data elements and ascertainment biases. Thus, for instance, TTE measurements performed at outside institutions were not available for these analyses. VUMC is a tertiary care center and may represent a sicker cohort than the general population. The data elements available for analysis are influenced by clinical decision making and practice patterns, which can lead to systematic biases. There was no active follow-up of study subjects, which can lead to differential censoring of data.

Finally, these analyses used a random forest algorithm to identify cases. While the threshold to define HF cases had PPV of 92%, up to 8% of cases may be misclassified. Importantly, the machine-learning algorithm heavily loaded on the frequency of instances that an individual was assigned billing codes for a heart failure diagnosis. Thus, the case definition is heavily based on the summative assessment of the clinical provider using clinical criteria relevant to the time period when the diagnosis was made. When cases used in the Validation set were evaluated using a Universal heart failure definition^20^, only 35% had sufficient data available in an electronic format to confirm that they met the case definition. Thus, the validity of the case definition with respect to a contemporary epidemiological case definition of heart failure is not well-defined. To fully address the robustness of the approach, it will need to be systematically evaluated in other native clinical environments.

In summary, we characterized the clinical epidemiology of HF subtypes derived from a large clinical data set. HF subtypes demonstrated distinct patterns of clinical co-morbidities and disease progression. Of direct clinical relevance, we demonstrate that, for HFpEF, these comorbidities are often not related to cardiac disease and manifest prior to the onset of a HF diagnosis. Awareness of these stereotypical patterns of clinical presentation will enhance early detection and prevention strategies, which is essential for HFpEF, which has no proven life-extending therapies.

## Supplementary Information

Supplementary Information.

## Data Availability

The aggregate data supporting the findings of this study are available from the corresponding author upon reasonable request; however, due to institutional data use agreements, access to individual level data is limited.

## Abbreviations

EHR: Electronic health record
ML: Machine learning
RF: Random forest
HF: Heart failure
HFpEF: Heart failure with preserved ejection fraction
HFrEF: Heart failure with reduced ejection fraction
HFmEF: Heart failure with mid-range ejection fraction
PPV: Positive predictive value
VUMC SD: Vanderbilt University Medical Center Synthetic Derivative
