# Supplementary material for: Heart failure clinical care analysis uncovers risk reduction opportunities for preserved ejection fraction subtype
Source: Sci Rep. 2021 Sep 20;11:18618. doi: 10.1038/s41598-021-97831-1 (PMC8452678; doi:10.1038/s41598-021-97831-1)
Supplement: Supplementary file 1 — Supplementary Information. [file 41598_2021_97831_MOESM1_ESM.pdf]

Supplementary Figures and Methods

Heart failure clinical care analysis uncovers risk reduction opportunities for preserved ejection fraction subtype

Rebecca T. Levinson, [$1,2] Nataraja Sarma Vaitinidin, *[$1] Eric Farber-Eger,[1] Dan M. Roden,[1,3,4]

Thomas A Lasko,[3] Quinn S. Wells, [$1,3,4] Jonathan D. Mosley, [$1,3]

[$]contributed equally, [*]corresponding author

Affiliations:

1) Department of Medicine, Vanderbilt University Medical Center, Nashville, TN, USA

2) Internal Medicine II, Heidelberg University Hospital, Heidelberg, Germany

3) Department of Biomedical Informatics, Vanderbilt University Medical Center, Nashville, TN, USA

4) Department of Pharmacology, Vanderbilt University Medical Center, Nashville, TN, USA

To whom correspondence should be addressed:

Nataraja Sarma Vaitinadin MBBS, PhD, MPH

Vanderbilt University Medical Center

1285 Medical Research Building IV

Nashville, TN 37232

Telephone: 615-322-0067

Fax: 615-343-4522

E-mail: nataraja.s.vaitinadin@vumc.org

Total word count ~3900

Supplementary figures referenced in the main text

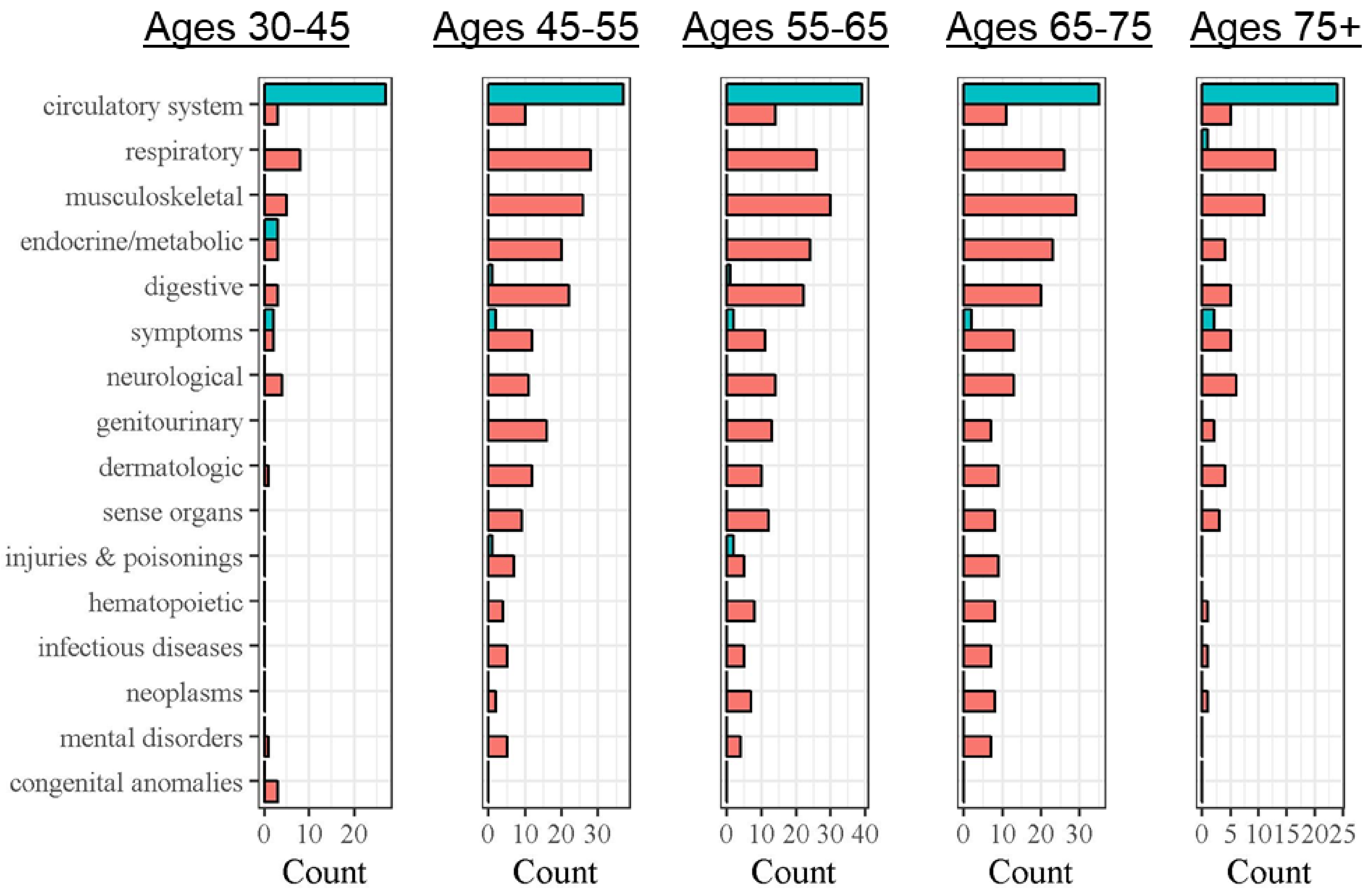

Supplementary Figure 1: Summary of PheWAS associations with HFpEF and HFrEF by disease category. The frequency histograms show the number of PheWAS phenotypes (assigned to pre-defined disease categories) with either a significantly higher prevalence in HFpEF (red bars) or HFrEF (blue bars). The figure shows that phenotypes more prevalent in HFrEF cases tend are predominantly related to the circulatory system, which phenotypes more prevalent in HFpEF fall in multiple disease categories.

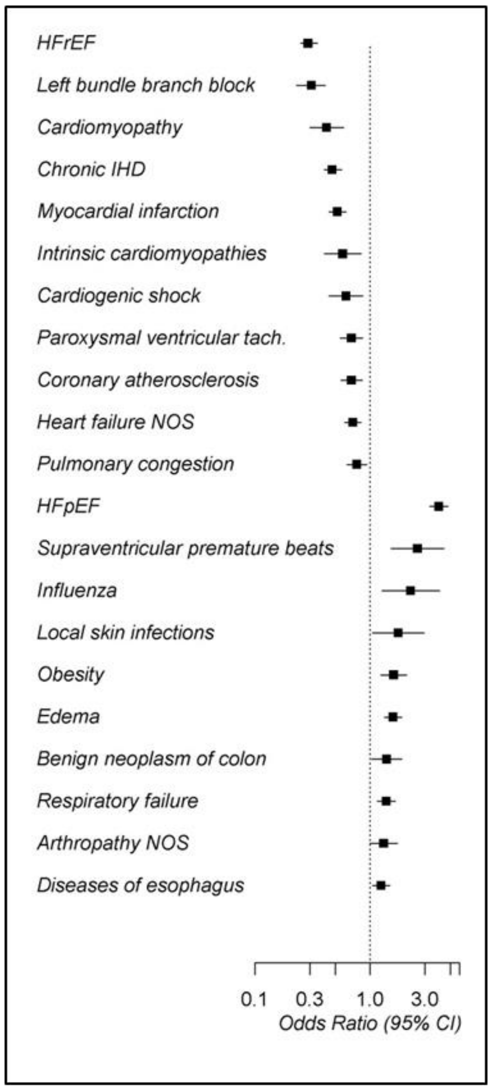

Supplementary Figure 2: Summary of PheWAS phenotypes associated with HFpEF versus HFrEF among individuals over 75 years old at the time of their TTE. Results for other age strata are shown in Figure 3.

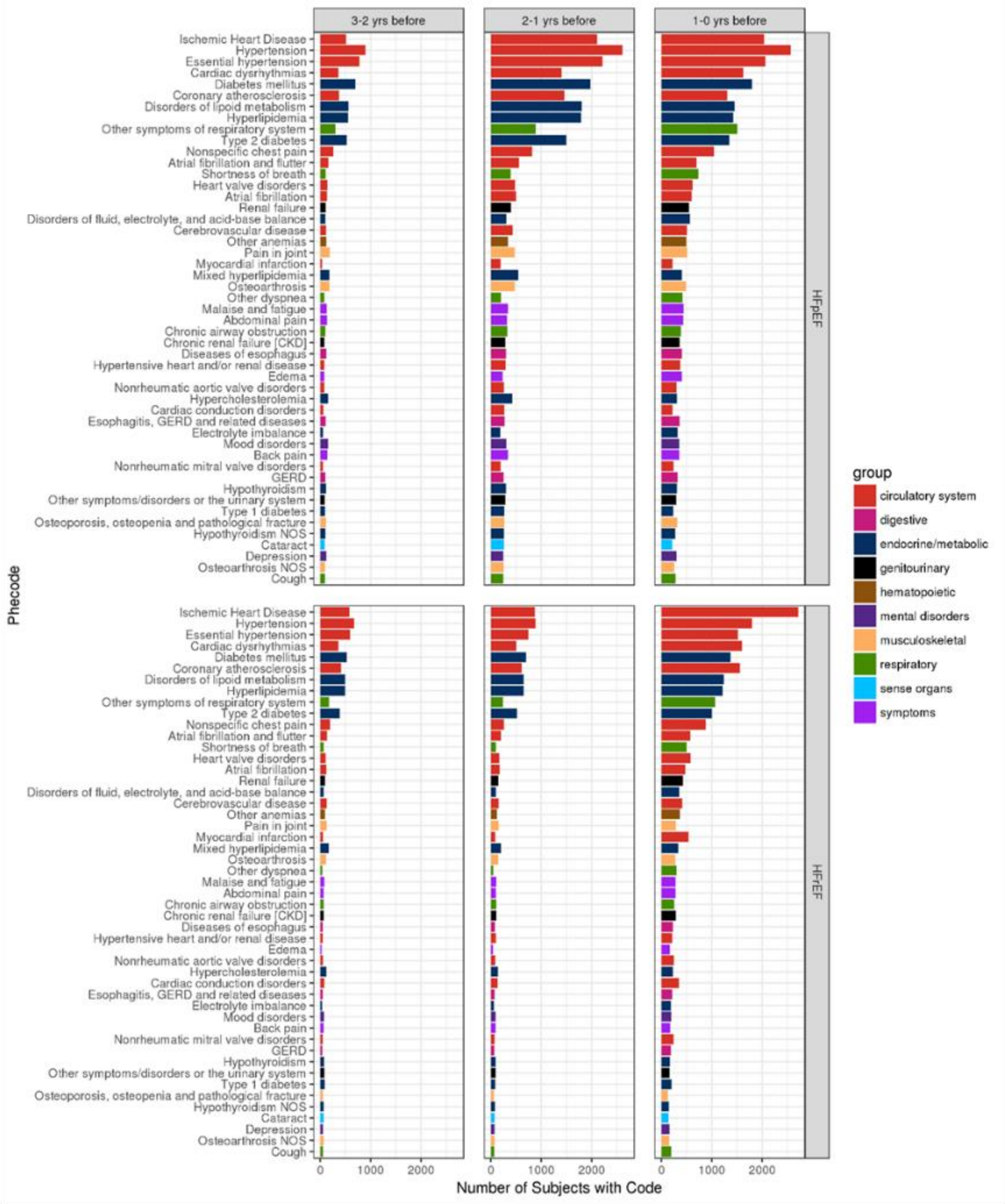

Supplementary Figure 3: A) Histogram of PheWAS codes in HFpEF and HFrEF patients prior to HF diagnosis. The frequency histograms show the number of HFpEF and HFrEF subjects with ICD9 codes mapping to each PheWAS phenotype in the three years prior to the onset of HF. PheWAS codes are groups into standard PheWAS code groups.

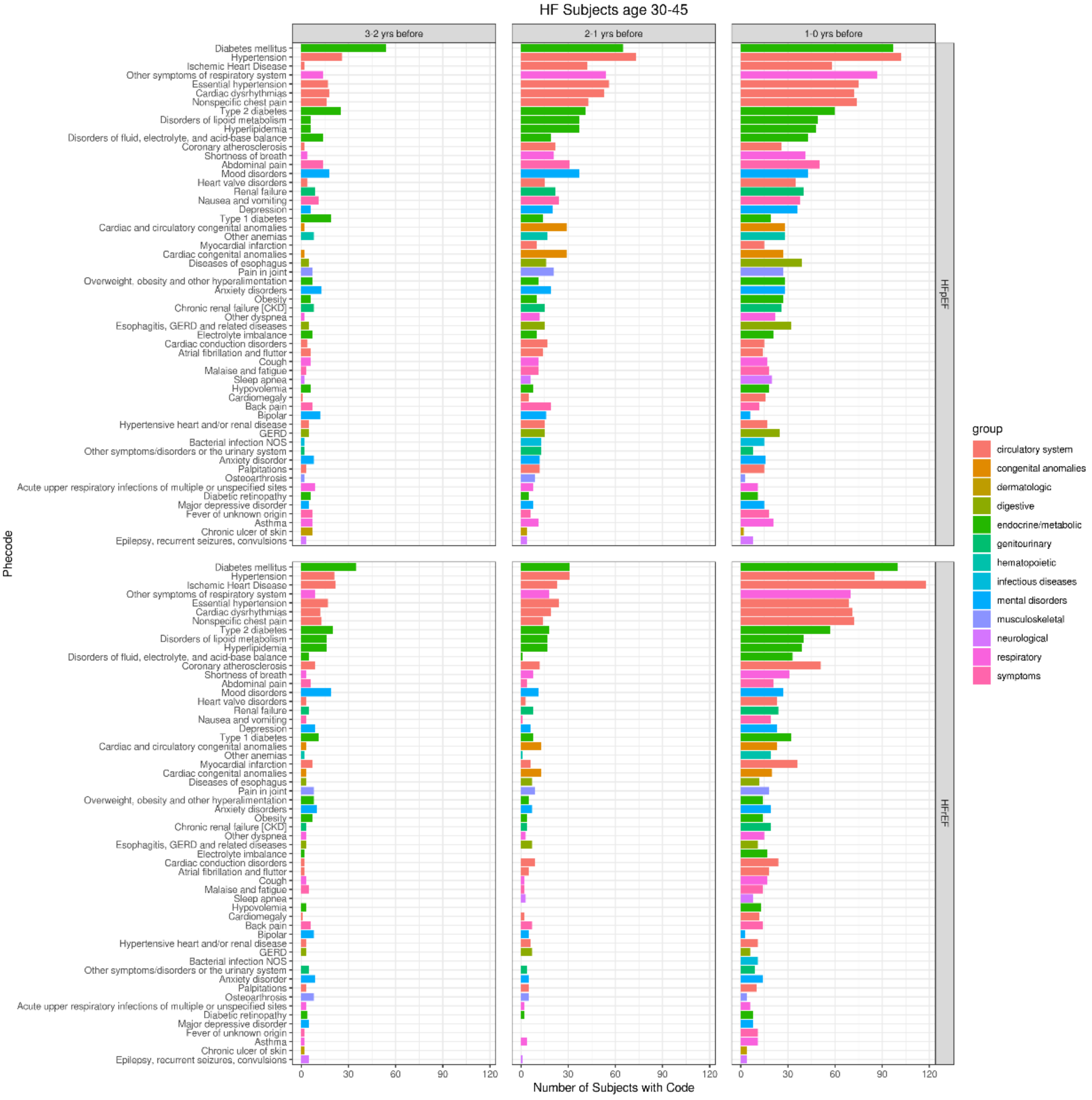

Supplementary Figure 3: B) Histogram of PheWAS codes in HFpEF and HFrEF patients prior to HF diagnosis, age 30-45 years.

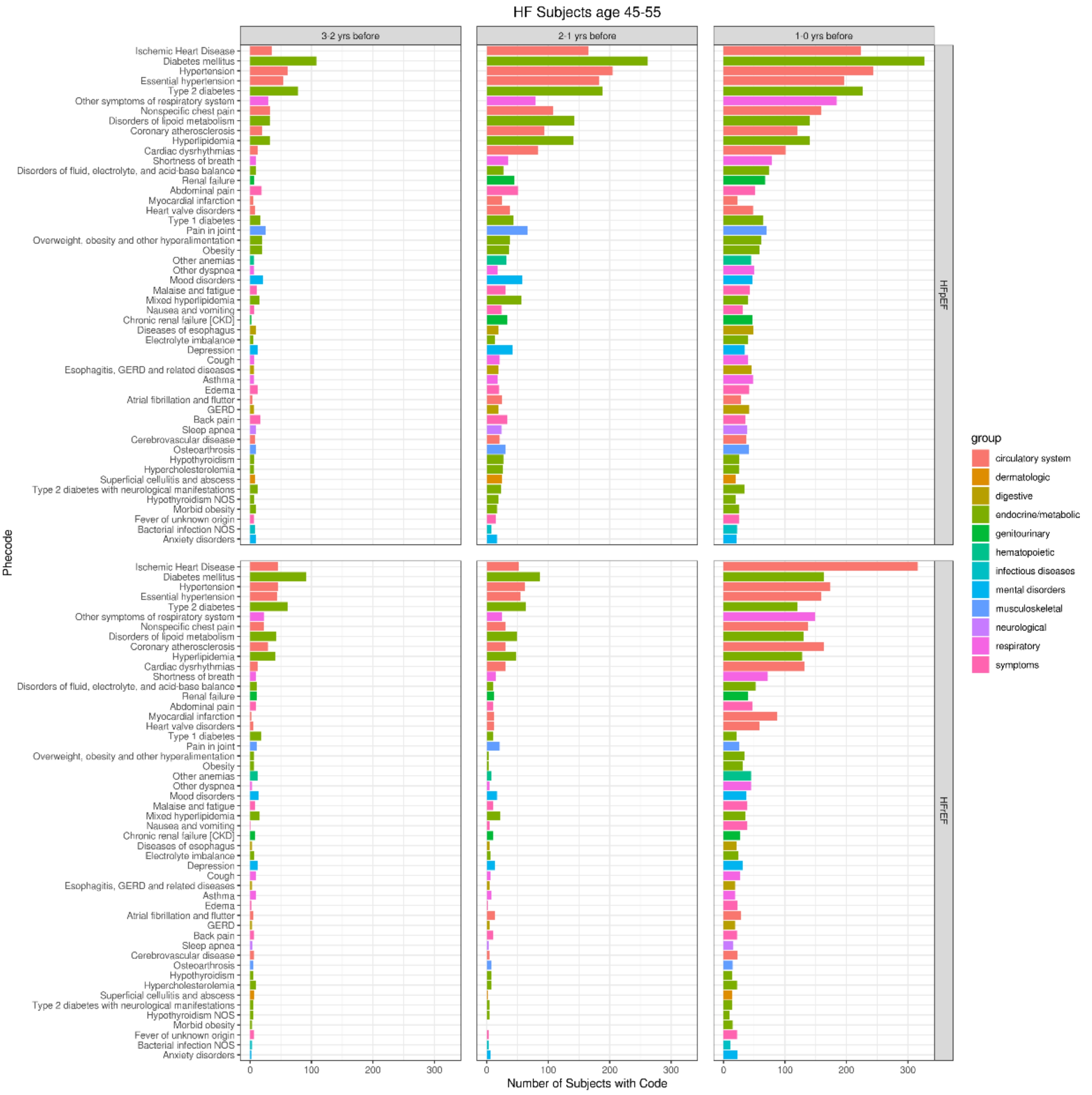

Supplementary Figure 3: C) Histogram of PheWAS codes in HFpEF and HFrEF patients prior to HF diagnosis, age 46-55 years.

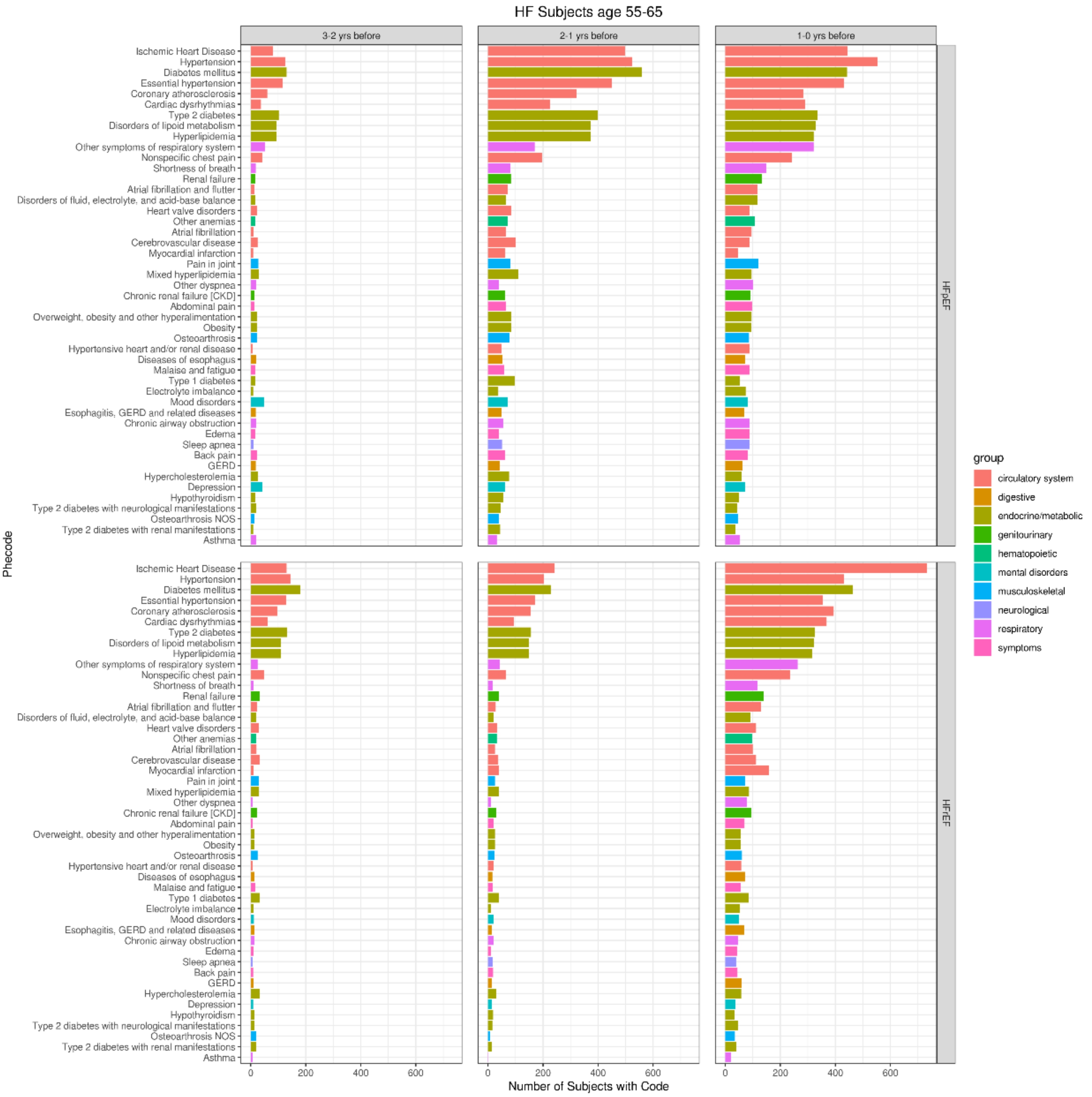

Supplementary Figure 3: D) Histogram of PheWAS codes in HFpEF and HFrEF patients prior to HF diagnosis, age 56-65 years.

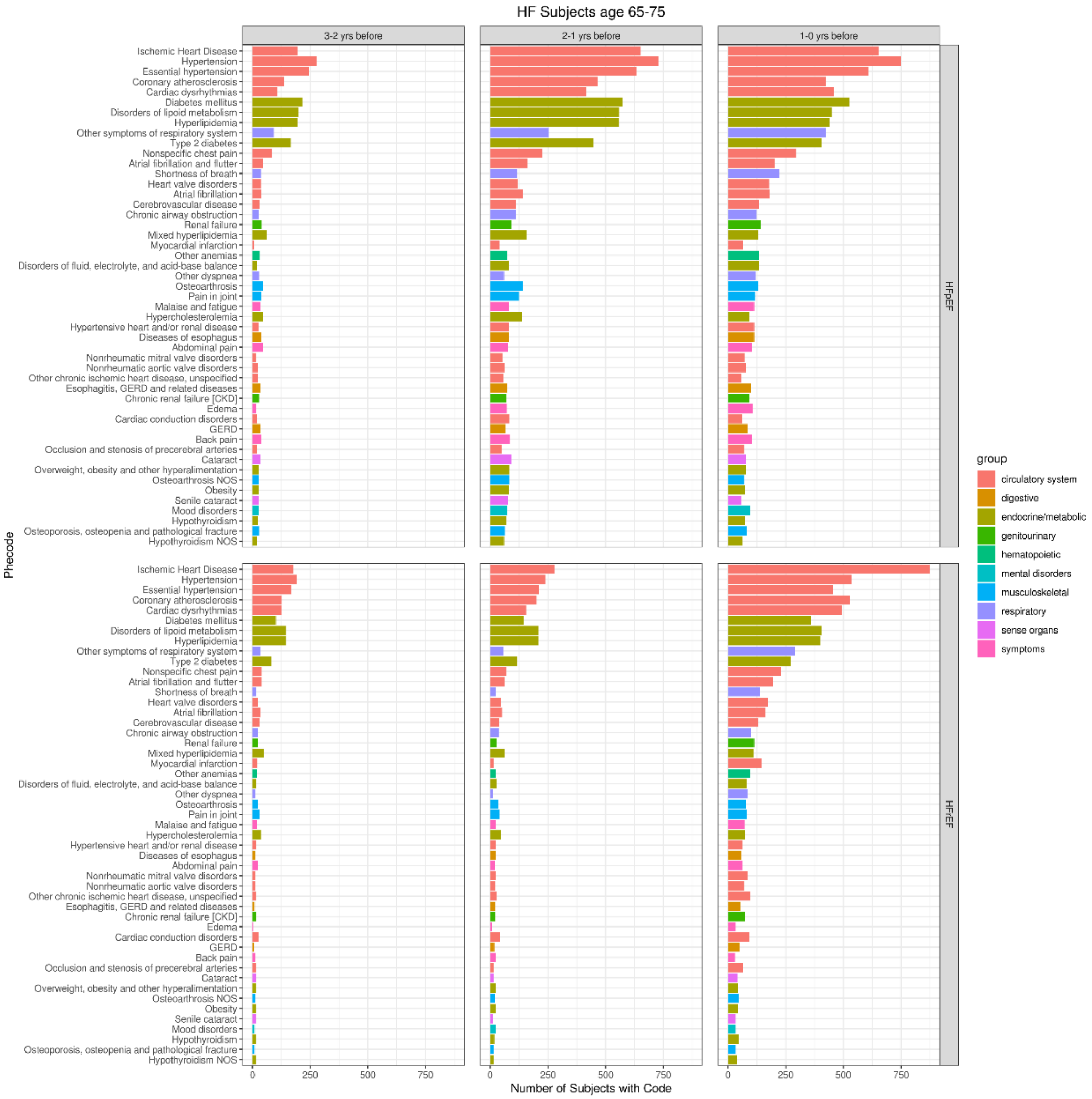

Supplementary Figure 3: E) Histogram of PheWAS codes in HFpEF and HFrEF patients prior to HF diagnosis, age 66-75 years.

Supplementary Methods

Study population

All subjects were derived from the Vanderbilt University Medical Center (VUMC) Synthetic Derivative (SD) database, a research tool for conducting epidemiological studies using de-identified clinical data. This resource comprises inpatient and outpatient clinical data from multiple sources including diagnostic and procedure codes (ICD-9 [International Classification of Disease, Ninth revision] and CPT [Current Procedural Terminology]), demographics, text from clinical notes, laboratory values, procedural reports e.g., echocardiograms), and medications extracted from ~2.7 million individual clinical records. The de-identified clinical records in the training and testing sets were manually reviewed by a clinical cardiologist and adjudicated to either a “Heart failure” or “No heart failure” group. For the training and testing sets there were 202 and 86 “Heart failure” subjects, respectively, and 889 and 382 “No heart failure” subjects.

Study Design

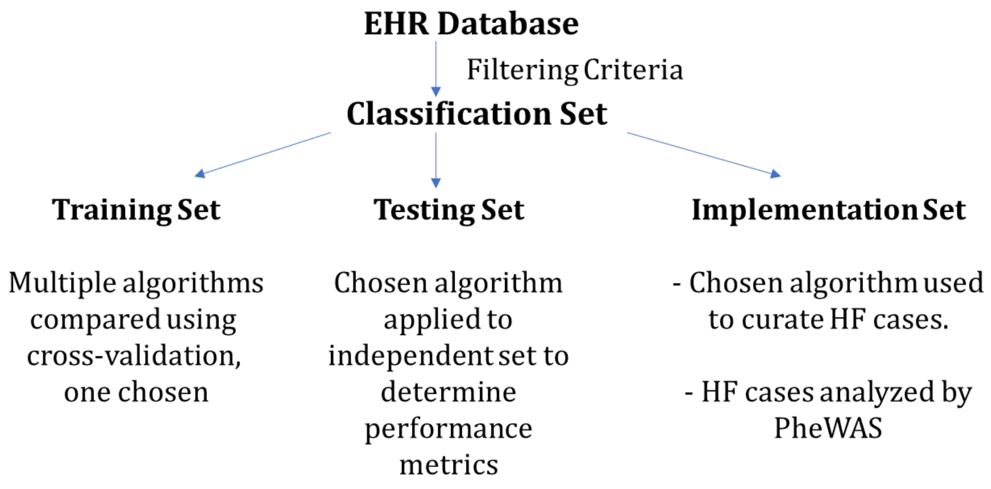

Supplementary Figure 4. Overview of the Random Forest implementation strategy.

Our goal was to identify prevalent adult HF cases in the EHR and define demographic and comorbid differences between HFpEF and HFrEF, that reflected current population trends. The initial pool of potential cases included individuals over 18 years old at their most recent clinical encounter with complete demographic information (gender, date-of-birth, race/ethnicity). There were fewer subjects with HF, in the order of tens of thousands, in our EHR database of ~2.7 million individuals. So, we further limited the source population to individuals meeting any of the following criteria: (1) they were assigned a HF-related ICD-9 diagnosis code (425* or 428*), (2) they had a non-negated mention of a HF-related term within any clinical document or (3) they had received a loop diuretic (n=674,560 individuals meeting these criteria) (see **Supplementary Tables 1 & 2** for list of search terms). A complete list of the data elements and phenotype definitions is presented in **Supplementary Table 3**. This approach improved learning by reducing class imbalance and removing cases with very limited evidence of HF, making classification trivial. From this group, randomly selected individuals were assigned to a training set (n=1,091) or a testing set (n=468). The remaining individuals were assigned to the implementation set (n=673,001). An overview of individual selection and set assignment is presented in **Supplementary Figure 4**.

Development of ML model

Machine learning features: Four categories of features were used for algorithm training: (1) keywords (text strings related to HF diagnoses or symptoms, New York Heart Association [NYHA] class assignments); (2) ICD-9 codes for HF (425*) or cardiomyopathy (428*); (3) medications (renin-angiotensin system inhibitors, beta blockers and loop diuretics); (4) lab values (B type natriuretic peptide [BNP] [coded as either >100 or >500 pg/m]); and (5) Other ICD-9 codes related to cardiovascular disease (390* to

459*). Medication data were extracted using the validated MEDEX tool. Keyword features were extracted from source documents (problem lists and clinical document) and occurrences were excluded if a negation term (such as “not” or “ruled out”) was within 100 characters of the keyword. We reasoned that features expressed in the EHR for longer periods of time and greater proportions of individual records were less likely to be false positives. Thus, we also derived the following metrics for each feature on a per subject basis and included them as separate features:

Feature strength = number of unique days with a mention

Feature persistence = number of days between first mention and last mention

Feature durability (percentage of record covered since time of first mention)

$$= \frac{(\#\ of\ days\ between\ first\ mention\ and\ last\ mention)}{(\#\ of\ days\ between\ first\ mention\ and\ last\ visit)}$$

Classifier creation: We developed a random forest machine learning HF classifier using the Python package *ScikitLearn* (v0.18.1). The random forest method iteratively constructs decision trees using an *a priori* set of defined features to segregate HF cases and non-cases in the training set. Feature weights were developed in the training set and then used to construct an individualized continuous predicted value (score) in the testing set, where the performance of the classifier was evaluated. Five-fold cross validation was used in the training set to optimize tree number and the number of features sampled for splitting at each node. A list of the final set of features selected and their relative importance is shown in **Supplementary Figure 5** and **Supplementary Table 4**.

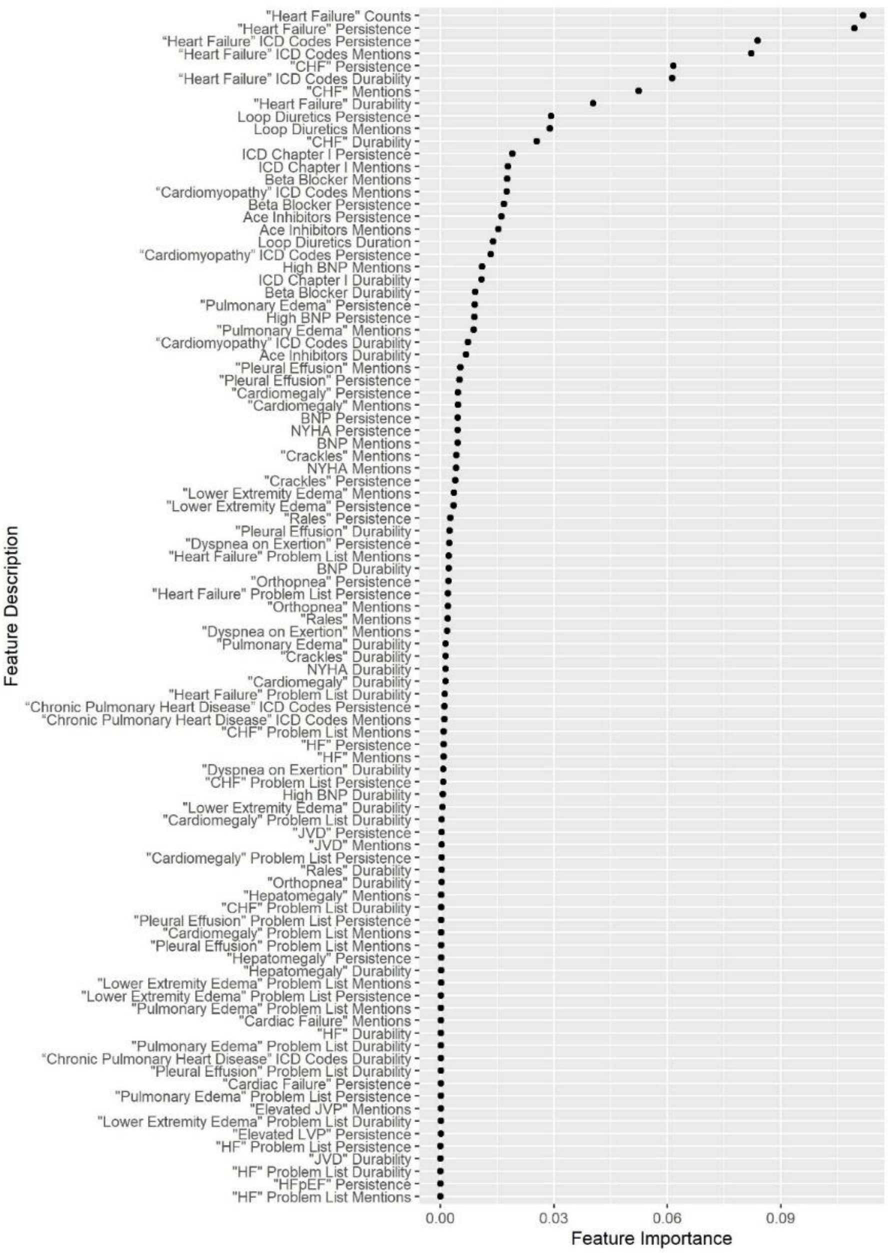

Supplementary Figure 5: Plot of importance of features with a non-zero importance score in the final random forest selected. Feature mentions are the total number of non-negated times a feature appears. Feature persistence is the number of days between first and last mention of a feature. Feature durability is the proportion of record after first feature mention in which the feature appears. All text features not specified as appearing in the problem list may appear anywhere else in the clinical record, features shown are with a non-zero estimate.

Comparison with other HF classification algorithms: Three additional algorithms were created, and their performance compared to the machine learning classifier in the testing set. Algorithm 1 identified HF cases as subjects with "heart failure", "chf", "hf", "hfpef", or "hfref" in their problem list. Algorithm 2 evaluated the presence of one or more 425* or 428* ICD-9 codes, a BNP >100 pg/ml, and a loop diuretic in a subject's record. Algorithm 3 was an EHR-based hierarchical classifier (i.e., case status defined as definite, probable, or possible) developed by the Electronic Medical Records and Genomics (eMERGE) network that uses ICD-9 code 428* and HF mentions from structured and unstructured problem lists.

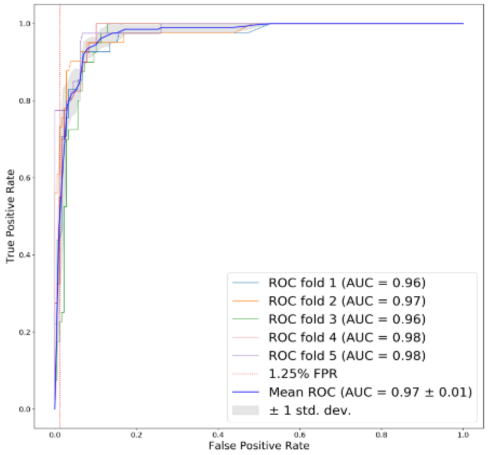

Supplementary Figure 6: AUC of random forest model among out-of-bag training samples and mean AUC across out-of-bag training samples. A false positive rate (FPR) of 1.25% used for case cut off in the testing set is shown with the red dotted line.

The model with the highest out-of-bag area under curve (AUC) was chosen as the optimal model. Using the mean receiver operating characteristic (ROC) curve in the out-of-bag training set, we chose the minimum score threshold that yielded a false positive rate (FPR) <1.25% as the

case score threshold for the testing and implementation sets. The final feature weightings were associated with an AUC of 0.97 in the training set (**Supplementary Figure 6**). Compared to other classification algorithms, the RF classifier had a slightly higher PPV (0.93 versus 0.88 for Algorithm 2) (**Supplementary Table 5**). Compared to the eMERGE algorithm, it had a lower sensitivity (0.67 versus 0.82) but higher PPV (0.92 vs 0.85) at the selected score threshold for case definition.

Implementation After model optimization, the predictor was applied to the testing set and a HF score was computed for every subject. Individuals with a score >0.732 (representing a 1.25% FPR in the training set) were considered HF cases, the rest were not considered as HF cases. This predictor was applied to the implementation set to classify cases using the same score threshold. The date of HF onset was defined as the first time point when a highly informative (based on random forest model feature weights) disease-defining heart failure feature (e.g., heart failure keyword, ICD code) appeared in the EHR. The optimized ML classifier had a positive predictive value (PPV) of 0.92, besides a case sensitivity of 0.67, when evaluated on the testing set.

Loop diuretic prescription

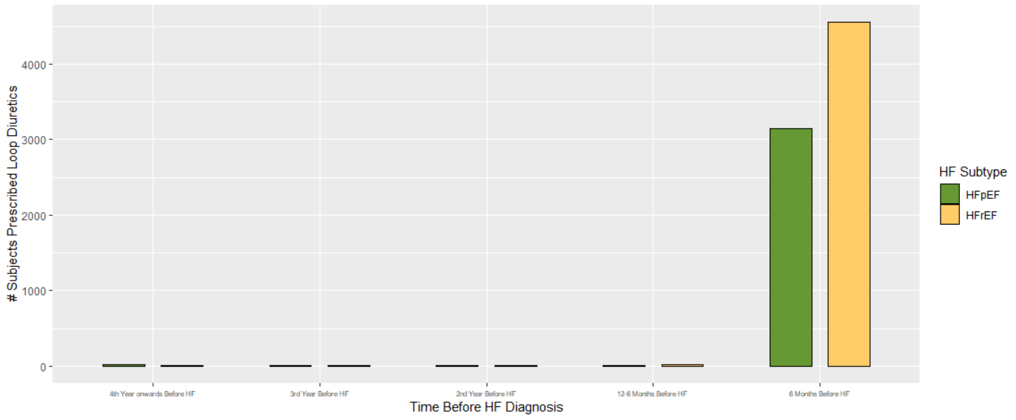

Supplementary Figure 7 Loop diuretic prescription mentions during the period before HF diagnosis, by HF subtype. (Refer **Supplementary Table 8**)

Supplementary Table 1: List of keywords used to select the initial population.

heart failure
cardiac failure
heart decompensation
cardiac insufficiency
myocardial failure
weak heart
hf
chf
hfpef
hfref
rales
crackles
elevated jugular venous pressure
elevated jvp
jvd
jugular venous distension
paroxysmal nocturnal dyspnea
orthopnea
third heart sound
cardiomegaly
pulmonary edema
lower extremity edema
dyspnea on exertion
pleural effusion
hepatomegaly
nocturnal cough

Supplementary Table 2: Heart Failure Random Forest Feature Database

Knowledge based feature selection. The terms in the feature database were collected and processed from the International Classification of Diseases, Ninth Revision, Clinical Modification (ICD-9-CM), Systematized Nomenclature of Medicine, Clinical Terms (SNOMED-CT), Medical Subject Headings (MeSH), symptoms from Framingham HF Diagnostic, and medications/labs associated with HF.

**Keywords**
**MeSH Derived**
Cardiac Failure
Heart Decompensation
Heart Failure, Right-Sided
Right-Sided Heart Failure
Myocardial Failure
Congestive Heart Failure
Heart Failure, Congestive
Heart Failure, Left-Sided
Left-Sided Heart Failure
**ICD-9 Derived**
Heart failure
Congestive heart failure
Left heart failure
Systolic heart failure
Acute systolic heart failure
Chronic systolic heart failure
Acute on chronic systolic heart failure
Diastolic heart failure
Acute diastolic heart failure
Chronic diastolic heart failure
Acute on chronic diastolic heart failure
Combined systolic and diastolic heart failure
Acute combined systolic and diastolic heart failure
Chronic combined systolic and diastolic heart failure

Acute on chronic combined systolic and diastolic heart failure
Heart failure
**SNOMED-CT (Concept: [84114007] - Heart failure) Derived (Synonyms)**
Cardiac failure (39482012)
Cardiac insufficiency (2969213019)
HF - Heart failure (1234906013)
Myocardial failure (139480016)
Weak heart (139481017)
**NYHA Class**
**Symptoms/Physical Findings (Framingham HF Criteria Derived)**
Rales
Crackles
Elevated jugular venous pressure
Elevated JVP
JVD
Jugular venous distension
Paroxysmal nocturnal dyspnea
Orthopnea
Third heart sound
Cardiomegaly
Pulmonary edema
Lower extremity edema
Dyspnea on exertion
Pleural effusion
Hepatomegally
Nocturnal cough
**Medications**
Loop diuretic
Angiotensin converting enzyme inhibitor/receptor blocker
Beta blocker
**Labs**
BNP

**ICD-9 codes**
425*
428*
390* to 459*

Supplementary Table 3. Definitions of algorithms used to define cohort clinical characteristics

Clinical Feature	Criteria
Hypertension	1 or more ICD Codes: 401.*-405.*<br>OR<br>Def 2: 1 or more Problem List: "hypertension", "htn" AND 0 in the Problem List: "portal hypertension", "pulmonary hypertension", "rv hypertension", "intracranial hypertension", "phtn"
Type 2 Diabetes	SEE https://phekb.org/phenotype/type-2-diabetes-demonstration-project
Coronary Artery Disease	2 or more ICD Codes: 410.*, 411.*, 412.*, 413.*, 414.*, V45.82<br>OR<br>1 or more CPT Codes: 33534-33536, 33510-33523, 92980-92982, 92984, 92995, 92996
Myocardial Infarction	1 or more ICD Codes: 410.*
Dyslipidemia	1 or more HDL < 40 (45 for female)<br>OR<br>1 or more Trigs > 200<br>OR<br>1 or more Cholesterol > 200<br>OR<br>1 or more of the following medications:<br>"atorvastatin","lipitor","torvast","lovastatin","altocor","pravastatin","pravachol","rosuvastatin",<br>"crestor","simvastatin","zocor","cholestyramine","prevalite","colestopil","colestid","colesevelam",<br>"welchol","niacin","niacor","niaspan","gemfibrozil","lopid","fenofibrate",<br>"tricor","fibrocor", "bezafibrate", "bezalip","ezetimibe","zetia"
Atrial Fibrillation	3 or more of the following:<br>"afib","a fib","atrial-fib","atrial fib","a flutter","atrial flutter","atrial-flutter" in the Problem List<br>"afib","a fib","atrial-fib","atrial fib","a flutter","atrial flutter","atrial-flutter" non-negated, non-family in other Clinical Documents
Chronic Kidney Disease	1 of more ICD Codes: 585.*

Supplementary Table 4: Feature importance in final random forest used for classification

Feature Descriptions	Feature Importance
"Heart Failure" Counts	0.111779146
"Heart Failure" Persistence	0.109441313
ICD9 428.* Persistence	0.083927107
ICD9 428.* Mentions	0.082178719
"CHF" Persistence	0.061595246
ICD9 428.* Durability	0.061295701
"CHF" Mentions	0.052378211
"Heart Failure" Durability	0.040383439
Loop Diuretics Persistence	0.029301643
Loop Diuretics Mentions	0.028918303
"CHF" Durability	0.025467448
ICD Chapter I Persistence	0.019022923
ICD Chapter I Mentions	0.017847714
Beta Blocker Mentions	0.017702378
ICD9 425.* Mentions	0.017566585
Beta Blocker Persistence	0.016832138
Ace Inhibitors Persistence	0.016152855
Ace Inhibitors Mentions	0.015278139
Loop Diuretics Duration	0.013946201
ICD9 425.* Persistence	0.013324813
High BNP Mentions	0.011000976
ICD Chapter I Durability	0.010902369
Beta Blocker Durability	0.009177756
"Pulmonary Edema" Persistence	0.009137467
High BNP Persistence	0.008988524
"Pulmonary Edema" Mentions	0.008819761
ICD9 425.* Durability	0.007271717
Ace Inhibitors Durability	0.006743121
"Pleural Effusion" Mentions	0.00533941
"Pleural Effusion" Persistence	0.005067788
"Cardiomegaly" Persistence	0.004620901
"Cardiomegaly" Mentions	0.004612957
BNP Persistence	0.00459944
NYHA Persistence	0.004592549
BNP Mentions	0.004546884
"Crackles" Mentions	0.004233897
NYHA Mentions	0.00408235
"Crackles" Persistence	0.00389218

"Lower Extremity Edema" Mentions	0.003608132
"Lower Extremity Edema" Persistence	0.003505346
"Rales" Persistence	0.002631557
"Pleural Effusion" Durability	0.002434331
"Dyspnea on Exertion" Persistence	0.002373012
"Heart Failure" Problem List Mentions	0.002254821
BNP Durability	0.002205288
"Orthopnea" Persistence	0.002131756
"Heart Failure" Problem List Persistence	0.002054894
"Orthopnea" Mentions	0.00199835
"Rales" Mentions	0.001911599
"Dyspnea on Exertion" Mentions	0.001840353
"Pulmonary Edema" Durability	0.001415195
"Crackles" Durability	0.001402327
NYHA Durability	0.001382567
"Cardiomegaly" Durability	0.00133085
"Heart Failure" Problem List Durability	0.001219713
ICD 416.* Persistence	0.001077224
ICD 416.* Mentions	0.001051052
"CHF" Problem List Mentions	0.000879145
"HF" Persistence	0.00086231
"HF" Mentions	0.000835164
"Dyspnea on Exertion" Durability	0.000719165
"CHF" Problem List Persistence	0.000699459
High BNP Durability	0.000618596
"Lower Extremity Edema" Durability	0.000563954
"Cardiomegaly" Problem List Durability	0.000369365
"JVD" Persistence	0.000306494
"JVD" Mentions	0.000294411
"Cardiomegaly" Problem List Persistence	0.000290766
"Rales" Durability	0.000290491
"Orthopnea" Durability	0.000286825
"Hepatomegaly" Mentions	0.000260554
"CHF" Problem List Durability	0.0002567
"Pleural Effusion" Problem List Persistence	0.000234653
"Cardiomegaly" Problem List Mentions	0.000220244
"Pleural Effusion" Problem List Mentions	0.000194569
"Hepatomegaly" Persistence	0.000182008
"Hepatomegaly" Durability	0.000179915
"Lower Extremity Edema" Problem List Mentions	0.000173355
"Lower Extremity Edema" Problem List Persistence	0.000162917

"Pulmonary Edema" Problem List Mentions	0.000140682
"Cardiac Failure" Mentions	0.00013927
"HF" Durability	0.000130411
"Pulmonary Edema" Problem List Durability	0.000108012
ICD 416.* Durability	0.000104212
"Pleural Effusion" Problem List Durability	0.000101912
"Cardiac Failure" Persistence	8.61716E-05
"Pulmonary Edema" Problem List Persistence	8.55157E-05
"Elevated JVP" Mentions	8.41045E-05
"Lower Extremity Edema" Problem List Durability	8.25237E-05
"Elevated LVP" Persistence	7.17018E-05
"HF" Problem List Persistence	4.78767E-05
"JVD" Durability	4.34916E-05
"HF" Problem List Durability	3.81102E-05
"HFpEF" Persistence	3.04251E-05
"HF" Problem List Mentions	2.60837E-05
"HFpEF" Mentions	0
"Cardiac Failure" Durability	0
"HFrEF" Mentions	0
"Noctural Cough" Problem List Persistence	0
"Noctural Cough" Problem List Mentions	0
"Noctural Cough" Problem List Durability	0
"Cardiac Failure" Problem List Mentions	0
"Heart Decompensation" Persistence	0
"Hepatomegaly" Problem List Durability	0
"Noctural Cough" Durability	0
"Noctural Cough" Persistence	0
"Noctural Cough" Mentions	0
"Hepatomegaly" Problem List Persistence	0
"Cardiac Failure" Problem List Persistence	0
"Hepatomegaly" Problem List Mentions	0
"Dyspnea on Exertion" Problem List Mentions	0
"Dyspnea on Exertion" Problem List Persistence	0
"Dyspnea on Exertion" Problem List Durability	0
"Heart Decompensation" Mentions	0
"Cardiac Failure" Problem List Durability	0
"Heart Decompensation" Durability	0
"Cardiac Insufficiency" Persistence	0
"Third Heart Sound" Problem List Durability	0
"HFrEF" Persistence	0
"HFpEF" Durability	0

"HFpEF" Problem List Mentions	0
"HFpEF" Problem List Persistence	0
"HFpEF" Problem List Durability	0
"HFrEF" Durability	0
"Heart Decompensation" Problem List Durability	0
"HFrEF" Problem List Mentions	0
"HFrEF" Problem List Persistence	0
"HFrEF" Problem List Durability	0
"Rales" Problem List Mentions	0
"Rales" Problem List Persistence	0
"Rales" Problem List Durability	0
"Heart Decompensation" Problem List Persistence	0
"Cardiac Insufficiency" Mentions	0
"Crackles" Problem List Persistence	0
"Myocardial Failure" Problem List Mentions	0
"Cardiac Insufficiency" Problem List Mentions	0
"Cardiac Insufficiency" Problem List Persistence	0
"Cardiac Insufficiency" Problem List Durability	0
"Myocardial Failure" Mentions	0
"Myocardial Failure" Persistence	0
"Myocardial Failure" Durability	0
"Myocardial Failure" Problem List Persistence	0
"Weak heart" Problem List Durability	0
"Myocardial Failure" Problem List Durability	0
"Weak heart" Mentions	0
"Weak heart" Persistence	0
"Weak heart" Durability	0
"Weak heart" Problem List Mentions	0
"Weak heart" Problem List Persistence	0
"Crackles" Problem List Mentions	0
"Crackles" Problem List Durability	0
"Third Heart Sound" Problem List Persistence	0
"Cardiac Insufficiency" Durability	0
"Paroxysmal Nocturnal Dyspnea" Mentions	0
"Paroxysmal Nocturnal Dyspnea" Persistence	0
"Paroxysmal Nocturnal Dyspnea" Durability	0
"Paroxysmal Nocturnal Dyspnea" Problem List Mentions	0
"Paroxysmal Nocturnal Dyspnea" Problem List Persistence	0
"Paroxysmal Nocturnal Dyspnea" Problem List Durability	0
"Orthopnea" Problem List Mentions	0
"Jugular Venous Distension" Problem List Persistence	0

"Orthopnea" Problem List Persistence	0
"Orthopnea" Problem List Durability	0
"Third Heart Sound" Mentions	0
"Third Heart Sound" Persistence	0
"Third Heart Sound" Durability	0
"Third Heart Sound" Problem List Mentions	0
"Jugular Venous Distension" Problem List Durability	0
"Heart Decompensation" Problem List Mentions	0
"Elevated Jugular Venous Pressure" Mentions	0
"Elevated JVP" Problem List Mentions	0
"Elevated Jugular Venous Pressure" Persistence	0
"Elevated Jugular Venous Pressure" Durability	0
"Elevated Jugular Venous Pressure" Problem List Mentions	0
"Elevated Jugular Venous Pressure" Problem List Persistence	0
"Elevated Jugular Venous Pressure" Problem List Durability	0
"Elevated JVP" Durability	0
"Elevated JVP" Problem List Persistence	0
"Jugular Venous Distension" Durability	0
"Elevated JVP" Problem List Durability	0
"JVP" Problem List Mentions	0
"JVP" Problem List Persistence	0
"JVP" Problem List Durability	0
"Jugular Venous Distension" Mentions	0
"Jugular Venous Distension" Persistence	0
"Jugular Venous Distension" Problem List Mentions	0

Supplementary Table 5: Heart Failure Random Forest Algorithm Comparison

Algorithm comparison evaluated the PPV, Sensitivity, Specificity, and NPV of the Random Forest and three other algorithms.

All comparisons are in the Testing set that acted as the hold out for the random forest building.

**Algorithm**	**PPV**	**Sensitivity**	**Specificity**	**NPV**
Random Forest	0.92	0.67	0.99	0.93
Algorithm 1 -HF in Problem List	0.87	0.23	0.99	0.85
Algorithm 2 -ICD9 Code, BNP >100, and Loop Diuretic	0.88	0.44	0.99	0.89
Algorithm 3 - eMERGE Algorithm	0.85	0.82	0.98	0.97

Supplementary Table 6: A) Characteristics of all HF cases and the HFpEF and HFrEF subtypes stratified by entry into care before/after 2005

	HF		HFpEF		HFrEF	
	**Before 2005**	**2005 and After**	**Before 2005**	**2005 and After**	**Before 2005**	**2005 and After**
**Characteristic ***	**(n=8681)**	**(n=19475)**	**(n=2566)**	**(n=5756)**	**(n=4076)**	**(n=7601)**
Male Gender [n(%)]	4665 (53.7)	11095 (57.0)	967 (37.7)	2521 (43.8)	2600 (63.8)	5106 (67.2)
Race/Ethnicity [n(%)]						
Black	1496 (17.2)	2743 (14.1)	493 (19.2)	821 (14.3)	726 (17.8)	1215 (16.0)
Other	529 (6.1)	1287 (6.6)	108 (4.2)	286 (5.0)	242 (5.9)	421 (5.5)
White	6656 (76.7)	15445 (79.3)	1965 (76.6)	4649 (80.8)	3108 (76.3)	5965 (78.5)
Age HF Entry	62.5 [51.7, 73.1]	66.0 [56.0, 75.3]	64.8 [53.2, 75.2]	67.3 [57.0, 76.7]	59.4 [49.3, 69.2]	63.1 [53.2, 72.4]
Age Last ICD9 Code (years)	68.8 [58.3, 78.7]	69.2 [59.2, 78.4]	71.3 [60.3, 81.6]	70.9 [60.6, 79.9]	66.4 [56.3, 76.1]	66.8 [56.7, 75.9]
Length of Record (years)	6.2 [1.8, 12.2]	5.2 [1.4, 10.2]	7.7 [3.0, 13.6]	6.4 [2.3, 11.8]	6.8 [2.3, 12.8]	5.5 [1.9, 10.3]
Median BP Systolic	122.0 [111.5, 133.0]	121.0 [111.0, 131.0]	128.0 [118.0, 138.0]	124.0 [115.5, 134.5]	118.0 [108.0, 129.0]	117.0 [107.0, 126.5]
Median BP Diastolic	68.0 [61.0, 74.0]	66.0 [60.0, 72.0]	68.0 [61.0, 74.0]	65.0 [59.0, 71.0]	67.0 [61.0, 73.0]	66.0 [60.0, 72.0]
Median Heart Rate	79.5 [71.5, 88.0]	76.0 [69.0, 84.0]	79.5 [72.0, 87.5]	75.5 [68.0, 84.0]	79.5 [71.5, 88.0]	76.5 [70.0, 85.0]
Median BMI	29.0 [24.7, 34.7]	29.4 [25.1, 35.0]	30.7 [25.5, 37.1]	30.7 [26.0, 36.9]	28.1 [24.4, 32.9]	28.2 [24.6, 323.0]
Myocardial infarct [n(%)]	2017 (23.2)	4616 (23.7)	484 (18.9)	1035 (18.0)	1217 (29.9)	2615 (34.4)
Coronary Artery Disease [n(%)]	5974 (68.8)	13130 (67.4)	1590 (62.0)	3616 (62.8)	3122 (76.6)	5836 (76.8)
Hypertension [n(%)]	7218 (83.1)	17345 (89.1)	2292 (89.3)	5308 (92.2)	3355 (82.3)	6847 (90.1)
Dyslipidemia [n(%)]	6567 (75.6)	16339 (83.9)	1937 (75.5)	4817 (83.7)	3319 (81.4)	6745 (88.7)
Type 2 diabetes [n(%)]	1593 (18.4)	4570 (23.5)	534 (20.8)	1417 (24.6)	759 (18.6)	1894 (24.9)
Atrial Fibrillation [n(%)]	3724 (42.9)	9168 (47.1)	1175 (45.8)	2803 (48.7)	1883 (46.2)	3821 (50.3)

Chronic Kidney Disease [n(%)]	2388 (27.5)	6733 (34.6)	755 (29.4)	2190 (38.0)	1276 (31.3)	3015 (39.7)
Lowest EF Value	35.0 [17.0, 55.0]	40.0 [22.5, 55.0]	55.0 [55.0, 55.0]	55.0 [55.0, 55.0]	20.0 [12.0, 30.0]	25.0 [15.0, 32.0]
BNP Measure Present [n(%)]	3144 (36.2)	13105 (67.3)	1042 (40.6)	4310 (74.9)	1732 (42.5)	5945 (78.2)
Median BNP Lifetime (pg/m)	362.3 [160.0, 820.0]	367.5 [142.0, 879.5]	268.8 [122.3, 589.8]	277.0 [106.0, 624.0]	465.5 [198.0, 1002.6]	487.0 [199.0, 1188.0]
Medical Home Status [n(%)]	5402 (62.2)	14040 (72.1)	1728 (67.3)	4271 (74.2)	2819 (69.2)	6106 (80.3)
Heart Failure Entry Year	2000 [1998, 2002]	2010 [2007, 2012]	2001 [1998, 2003]	2010 [2008, 2012]	2000 [1998, 2002]	2010 [2007, 2012]

Supplementary Table 6: B) Fraction of Comorbid associations before and after HF diagnosis, by HF subtype.

HF Subtype	Group	After HF	Before HF	Fraction Before HF
HFpEF	HTN	2413	1865	0.436
HFrEF		4511	1989	0.306
HFpEF	DysLip	2377	1486	0.3847
HFrEF		4623	1813	0.2817
HFpEF	CAD	2214	846	0.2765
HFrEF		4404	1277	0.2248
HFpEF	MI	873	140	0.1382
HFrEF		2357	301	0.1132
HFpEF	emerge DM	932	251	0.2122
HFrEF		1582	321	0.1687
HFpEF	DM	1770	914	0.3405
HFrEF		2996	1067	0.2626
HFpEF	CKD	1891	206	0.0982
HFrEF		3090	216	0.0653

Supplementary Table 7: PheWAS phenotype associations from the forward selection models.

Results are stratified by age range.

Age range	PheWAS code	PheWAS phenotype	Cases	Controls	Odds ratio	95% CI	p-value
30-45	428.4	Heart failure with preserved EF [Diastolic heart failure]	425	1479	3.44	(2.50-4.73)	3.9E-14
30-45	428.3	Heart failure with reduced EF [Systolic or combined heart failure	1024	868	0.35	(0.26-0.46)	4.1E-14
30-45	327.3	Sleep apnea	505	1389	2.43	(1.79-3.29)	1.0E-08
30-45	411.8	Other chronic ischemic heart disease, unspecified	595	1241	0.47	(0.33-0.67)	3.0E-05
30-45	411.2	Myocardial infarction	546	1290	0.5	(0.35-0.69)	4.0E-05
30-45	396	Abnormal heart sounds	186	1718	2.04	(1.37-3.04)	4.8E-04
30-45	747.11	Cardiac shunt/ heart septal defect	156	1748	2.14	(1.35-3.40)	1.3E-03
30-45	428.2	Heart failure NOS	1075	829	0.65	(0.50-0.85)	1.7E-03
30-45	426.92	Cardiac defibrillator in situ	552	1341	0.52	(0.34-0.79)	2.0E-03
30-45	797.1	Cardiogenic shock	250	1654	0.47	(0.29-0.77)	2.3E-03
30-45	415.21	Primary pulmonary hypertension	322	1582	1.68	(1.20-2.35)	2.7E-03
30-45	340	Migraine	164	1728	1.89	(1.20-2.97)	5.9E-03
30-45	550	Abdominal hernia	215	1689	1.76	(1.16-2.66)	7.3E-03
30-45	425.1	Primary/intrinsic cardiomyopathies	1275	629	0.41	(0.21-0.80)	8.9E-03
30-45	429.1	Heart transplant/surgery	323	1570	0.59	(0.39-0.89)	1.1E-02
30-45	425.2	Secondary/extrinsic cardiomyopathies	541	1352	0.65	(0.45-0.93)	1.9E-02

30-45	426.32	Left bundle branch block	191	1713	0.47	(0.25-0.90)	2.2E-02
30-45	426.91	Cardiac pacemaker in situ	357	1547	1.63	(1.05-2.54)	3.0E-02
30-45	425	Cardiomyopathy	1336	568	0.49	(0.24-1.00)	5.0E-02

Age range	PheWAS code	PheWAS phenotype	Cases	Controls	Odds ratio	95% CI	p-value
45-55	428.4	Heart failure with preserved EF [Diastolic heart failure]	854	2360	4.13	(3.21-5.32)	3.4E-28
45-55	425.1	Primary/intrinsic cardiomyopathies	1977	1237	0.31	(0.25-0.38)	5.9E-27
45-55	428.3	Heart failure with reduced EF [Systolic or combined heart failure	1650	1551	0.33	(0.26-0.41)	1.6E-23
45-55	411.2	Myocardial infarction	1258	1956	0.45	(0.36-0.57)	5.1E-11
45-55	411.8	Other chronic ischemic heart disease, unspecified	1403	1811	0.47	(0.37-0.59)	1.6E-10
45-55	428.2	Heart failure NOS	1727	1487	0.56	(0.45-0.69)	5.6E-08
45-55	426.32	Left bundle branch block	412	2802	0.25	(0.14-0.46)	7.0E-06
45-55	278.11	Morbid obesity	699	2504	1.78	(1.38-2.30)	9.1E-06
45-55	426.92	Cardiac defibrillator in situ	910	2291	0.49	(0.36-0.68)	2.0E-05
45-55	401	Hypertension	2752	462	1.88	(1.40-2.53)	2.8E-05
45-55	427.7	Tachycardia NOS	1120	2094	0.61	(0.49-0.77)	3.3E-05
45-55	428.1	Congestive heart failure (CHF) NOS	2998	216	0.5	(0.34-0.74)	4.5E-04
45-55	512	Other symptoms of respiratory system	2608	606	1.62	(1.23-2.14)	6.6E-04
45-55	425.2	Secondary/extrinsic cardiomyopathies	895	2306	0.64	(0.49-0.83)	6.9E-04
45-55	585.32	End stage renal disease	351	2851	1.94	(1.30-2.88)	1.1E-03
45-55	502	Postinflammatory pulmonary fibrosis	155	3059	2.18	(1.36-3.49)	1.1E-03
45-55	495	Asthma	489	2725	0.61	(0.46-0.83)	1.3E-03
45-55	440.22	Atherosclerosis of native arteries of the extremities with interm	213	2998	0.5	(0.31-0.79)	3.0E-03
45-55	797.1	Cardiogenic shock	388	2826	0.59	(0.41-0.84)	3.7E-03

45-55	530	Diseases of esophagus	1088	2126	1.39	(1.11-1.75)	4.1E-03
45-55	578.8	Hemorrhage of rectum and anus	157	3057	2	(1.24-3.22)	4.3E-03
45-55	418.1	Precordial pain	377	2837	1.61	(1.16-2.23)	4.4E-03
45-55	496	Chronic airway obstruction	1038	2176	1.38	(1.10-1.74)	5.5E-03
45-55	427.6	Premature beats	380	2834	0.63	(0.45-0.88)	7.0E-03
45-55	782.3	Edema	1066	2148	1.36	(1.08-1.72)	9.5E-03
45-55	285.21	Anemia in chronic kidney disease	283	2918	0.56	(0.36-0.87)	1.1E-02
45-55	276.6	Fluid overload	898	2316	1.39	(1.08-1.79)	1.1E-02
45-55	509.8	Dependence on respirator [Ventilator] or Supplementary oxygen	320	2894	1.53	(1.07-2.19)	2.1E-02
45-55	770	Myalgia and myositis unspecified	351	2863	1.49	(1.06-2.10)	2.2E-02
45-55	395.1	Nonrheumatic mitral valve disorders	1332	1882	0.78	(0.63-0.96)	2.2E-02
45-55	426.91	Cardiac pacemaker in situ	597	2617	1.43	(1.04-1.96)	3.0E-02
45-55	426.3	Bundle branch block	556	2658	1.64	(1.02-2.65)	4.1E-02

**Age range**	**PheWAS code**	**PheWAS phenotype**	**Cases**	**Controls**	**Odds ratio**	**95% CI**	**p-value**
55-65	428.4	Heart failure with preserved EF [Diastolic heart failure]	1372	3616	4.22	(3.48-5.13)	3.2E-48
55-65	428.3	Heart failure with reduced EF [Systolic or combined heart failure	2596	2360	0.31	(0.26-0.37)	7.0E-39
55-65	411.8	Other chronic ischemic heart disease, unspecified	2487	2501	0.52	(0.43-0.62)	6.8E-13
55-65	426.92	Cardiac defibrillator in situ	1393	3564	0.4	(0.31-0.52)	2.1E-12
55-65	411.2	Myocardial infarction	2276	2712	0.56	(0.46-0.67)	2.3E-10
55-65	426.32	Left bundle branch block	636	4352	0.38	(0.27-0.52)	8.7E-09
55-65	428.2	Heart failure NOS	2495	2493	0.61	(0.52-0.72)	1.0E-08
55-65	395.1	Nonrheumatic mitral valve disorders	2212	2776	0.6	(0.51-0.72)	1.4E-08
55-65	797.1	Cardiogenic shock	557	4431	0.5	(0.37-0.68)	7.0E-06
55-65	401.1	Essential hypertension	4248	740	1.61	(1.27-2.02)	5.9E-05
55-65	278.11	Morbid obesity	881	4076	1.6	(1.27-2.01)	6.9E-05
55-65	276.6	Fluid overload	1352	3636	1.47	(1.20-1.79)	1.6E-04
55-65	425.1	Primary/intrinsic cardiomyopathies	2690	2298	0.49	(0.34-0.71)	1.9E-04
55-65	425	Cardiomyopathy	2893	2095	0.51	(0.35-0.74)	3.4E-04
55-65	366.2	Senile cataract	628	4360	0.61	(0.47-0.80)	3.9E-04
55-65	394.7	Disease of tricuspid valve	959	4029	1.44	(1.15-1.82)	1.6E-03
55-65	427.41	Ventricular fibrillation and flutter	395	4593	0.5	(0.32-0.77)	1.8E-03
55-65	327.3	Sleep apnea	1282	3676	1.37	(1.11-1.69)	3.4E-03
55-65	714.1	Rheumatoid arthritis	232	4756	1.75	(1.20-2.54)	3.5E-03

55-65	783	Fever of unknown origin	1520	3468	0.75	(0.62-0.92)	5.0E-03
55-65	440.2	Atherosclerosis of the extremities	662	4326	0.71	(0.55-0.91)	7.1E-03
55-65	327.4	Insomnia	463	4525	1.49	(1.11-1.99)	7.4E-03
55-65	509	Respiratory failure, insufficiency, arrest	2042	2946	1.28	(1.06-1.54)	1.0E-02
55-65	285	Other anemias	2537	2451	1.27	(1.05-1.54)	1.2E-02
55-65	571.51	Cirrhosis of liver without mention of alcohol	243	4745	1.61	(1.11-2.34)	1.2E-02
55-65	401.21	Hypertensive heart disease	1313	3675	1.28	(1.05-1.56)	1.5E-02
55-65	972.1	Cardiac rhythm regulators causing adverse effects in therapeutic	129	4859	0.5	(0.27-0.92)	2.6E-02
55-65	415.11	Pulmonary embolism and infarction, acute	281	4707	1.49	(1.05-2.12)	2.7E-02
55-65	530	Diseases of esophagus	1767	3221	1.23	(1.02-1.47)	2.8E-02
55-65	562	Diverticulosis and diverticulitis	764	4224	1.29	(1.02-1.64)	3.5E-02
55-65	743.2	Pathologic fracture	138	4850	1.66	(1.03-2.68)	3.8E-02
55-65	374	Other disorders of eyelids	183	4805	1.59	(1.02-2.48)	4.1E-02
55-65	720	Spinal stenosis	325	4663	1.42	(1.01-1.98)	4.2E-02

**Age range**	**PheWAS code**	**PheWAS phenotype**	**Cases**	**Controls**	**Odds ratio**	**95% CI**	**p-value**
65-75	428.4	Heart failure with preserved EF [Diastolic heart failure]	1625	3424	4.05	(3.36-4.87)	1.1E-49
65-75	428.3	Heart failure with reduced EF [Systolic or combined heart failure	2510	2508	0.3	(0.25-0.36)	2.4E-43
65-75	411.8	Other chronic ischemic heart disease, unspecified	2403	2646	0.47	(0.39-0.56)	3.1E-16
65-75	426.32	Left bundle branch block	691	4358	0.32	(0.23-0.44)	6.0E-13
65-75	425.11	Hypertrophic obstructive cardiomyopathy	111	4938	7.21	(4.20-12.40)	7.6E-13
65-75	411.2	Myocardial infarction	2218	2831	0.59	(0.49-0.70)	4.1E-09
65-75	797	Shock	825	4224	0.53	(0.42-0.67)	1.6E-07
65-75	278	Overweight, obesity and other hyperalimentation	1323	3696	1.64	(1.35-2.00)	6.6E-07
65-75	425	Cardiomyopathy	2471	2578	0.44	(0.31-0.61)	9.9E-07
65-75	428.2	Heart failure NOS	2314	2735	0.67	(0.57-0.79)	2.4E-06
65-75	427.12	Paroxysmal ventricular tachycardia	1420	3629	0.6	(0.48-0.75)	9.3E-06
65-75	395.1	Nonrheumatic mitral valve disorders	2277	2772	0.7	(0.59-0.82)	1.9E-05
65-75	389	Hearing loss	509	4540	1.73	(1.32-2.29)	9.3E-05
65-75	430.1	Subarachnoid hemorrhage	255	4794	0.46	(0.31-0.68)	9.6E-05
65-75	512	Other symptoms of respiratory system	4183	866	1.49	(1.19-1.86)	4.2E-04
65-75	426.92	Cardiac defibrillator in situ	1232	3786	0.62	(0.48-0.82)	5.5E-04
65-75	741.3	Difficulty in walking	238	4811	0.5	(0.33-0.75)	9.9E-04
65-75	288.2	Elevated white blood cell count	691	4326	1.5	(1.17-1.92)	1.2E-03
65-75	401.1	Essential hypertension	4383	666	1.48	(1.16-1.88)	1.4E-03

65-75	509.8	Dependence on respirator [Ventilator] or Supplementary oxygen	514	4535	1.53	(1.16-2.01)	2.4E-03
65-75	425.1	Primary/intrinsic cardiomyopathies	2209	2840	0.59	(0.42-0.84)	3.2E-03
65-75	276.6	Fluid overload	1135	3914	1.35	(1.11-1.66)	3.2E-03
65-75	426.7	Abnormal electrocardiogram [ECG] [EKG]	1048	4001	0.75	(0.61-0.91)	3.9E-03
65-75	270.3	Disorders of plasma protein metabolism	277	4772	1.63	(1.17-2.28)	4.1E-03
65-75	427.8	Sinoatrial node dysfunction (Bradycardia)	925	4124	1.37	(1.10-1.71)	5.6E-03
65-75	480	Pneumonia	1695	3354	0.77	(0.65-0.93)	6.1E-03
65-75	809	Fracture of unspecified bones	173	4876	1.87	(1.19-2.93)	6.2E-03
65-75	782.3	Edema	1676	3373	1.29	(1.07-1.54)	7.1E-03
65-75	504	Other alveolar and parietoalveolar pneumonopathy	129	4920	2.03	(1.18-3.49)	1.1E-02
65-75	571	Chronic liver disease and cirrhosis	397	4620	1.46	(1.08-1.96)	1.4E-02
65-75	766	Neuralgia, neuritis, and radiculitis NOS	199	4850	1.69	(1.10-2.60)	1.8E-02
65-75	370	Keratitis	139	4910	1.85	(1.11-3.10)	1.9E-02
65-75	327.3	Sleep apnea	996	4022	1.29	(1.04-1.61)	2.3E-02
65-75	285	Other anemias	2612	2437	1.22	(1.03-1.46)	2.5E-02
65-75	599.4	Urinary incontinence	381	4668	1.39	(1.02-1.89)	3.9E-02
65-75	411	Ischemic Heart Disease	4157	892	0.78	(0.62-0.99)	3.9E-02
65-75	427.42	Cardiac arrest	358	4691	0.71	(0.51-0.98)	4.0E-02

**Age range**	**PheWAS code**	**PheWAS phenotype**	**Cases**	**Controls**	**Odds ratio**	**95% CI**	**p-value**
>75	428.4	Heart failure with preserved EF [Diastolic heart failure]	1508	2809	3.98	(3.32-4.78)	7.5E-50
>75	428.3	Heart failure with reduced EF [Systolic or combined heart failure	1784	1913	0.29	(0.25-0.35)	4.9E-46
>75	411.8	Other chronic ischemic heart disease, unspecified	1615	2702	0.47	(0.40-0.57)	5.4E-16
>75	426.32	Left bundle branch block	500	3817	0.31	(0.23-0.41)	2.3E-15
>75	411.2	Myocardial infarction	1696	2621	0.52	(0.44-0.62)	2.6E-13
>75	782.3	Edema	1601	2716	1.59	(1.33-1.90)	2.6E-07
>75	425	Cardiomyopathy	1547	2770	0.42	(0.30-0.59)	7.2E-07
>75	428.2	Heart failure NOS	1562	2755	0.71	(0.60-0.84)	1.0E-04
>75	509	Respiratory failure, insufficiency, arrest	1426	2891	1.39	(1.16-1.67)	3.1E-04
>75	278.1	Obesity	519	3165	1.61	(1.24-2.09)	3.7E-04
>75	427.61	Supraventricular premature beats	110	4207	2.59	(1.53-4.41)	4.3E-04
>75	411.4	Coronary atherosclerosis	3237	1080	0.69	(0.56-0.86)	6.4E-04
>75	427.12	Paroxysmal ventricular tachycardia	745	3572	0.69	(0.55-0.87)	1.9E-03
>75	425.1	Primary/intrinsic cardiomyopathies	1331	2986	0.58	(0.40-0.84)	3.5E-03
>75	481	Influenza	101	4216	2.26	(1.27-4.03)	5.7E-03
>75	797.1	Cardiogenic shock	273	4044	0.62	(0.44-0.87)	6.0E-03
>75	503	Pulmonary congestion and hypostasis	993	3324	0.77	(0.63-0.94)	9.4E-03
>75	530	Diseases of esophagus	1428	2889	1.25	(1.05-1.49)	1.4E-02
>75	208	Benign neoplasm of colon	351	3966	1.4	(1.03-1.90)	3.0E-02

>75	686	Other local infections of skin and subcutaneous tissue	140	4177	1.76	(1.05-2.95)	3.1E-02
>75	716.9	Arthropathy NOS	481	3836	1.32	(1.00-1.73)	4.7E-02

Supplementary Table 8. Loop diuretic prescription mentions, as %, during the period before HF diagnosis, by HF subtype.

HF Subtype	Total	4th Year onwards Before HF	3rd Year Before HF	2nd Year Before HF	12-6 Months Before HF	6 Months Before HF
HFpEF	3197	0.59	0.13	0.41	0.44	98.44
HFrEF	4601	0.24	0.24	0.17	0.35	99.00
